# Digital Reconstruction of the Neuro-Glia-Vascular Architecture

**DOI:** 10.1093/cercor/bhab254

**Published:** 2021-08-13

**Authors:** Eleftherios Zisis, Daniel Keller, Lida Kanari, Alexis Arnaudon, Michael Gevaert, Thomas Delemontex, Benoît Coste, Alessandro Foni, Marwan Abdellah, Corrado Calì, Kathryn Hess, Pierre Julius Magistretti, Felix Schürmann, Henry Markram

**Affiliations:** Blue Brain Project, École polytechnique fédérale de Lausanne (EPFL), Campus Biotech, Geneva 1202, Switzerland; Blue Brain Project, École polytechnique fédérale de Lausanne (EPFL), Campus Biotech, Geneva 1202, Switzerland; Blue Brain Project, École polytechnique fédérale de Lausanne (EPFL), Campus Biotech, Geneva 1202, Switzerland; Blue Brain Project, École polytechnique fédérale de Lausanne (EPFL), Campus Biotech, Geneva 1202, Switzerland; Blue Brain Project, École polytechnique fédérale de Lausanne (EPFL), Campus Biotech, Geneva 1202, Switzerland; Blue Brain Project, École polytechnique fédérale de Lausanne (EPFL), Campus Biotech, Geneva 1202, Switzerland; Blue Brain Project, École polytechnique fédérale de Lausanne (EPFL), Campus Biotech, Geneva 1202, Switzerland; Blue Brain Project, École polytechnique fédérale de Lausanne (EPFL), Campus Biotech, Geneva 1202, Switzerland; Blue Brain Project, École polytechnique fédérale de Lausanne (EPFL), Campus Biotech, Geneva 1202, Switzerland; Neuroscience Institute Cavalieri Ottolenghi, Orbassano, Turin 10043, Italy; Department of Neuroscience, University of Torino, Torino 10126, Italy; Biological and Environmental Sciences and Engineering Division, King Abdullah University of Science and Technology (KAUST), Thuwal 23955, Saudi Arabia; Laboratory for Topology and Neuroscience, Brain Mind Institute, École polytechnique fédérale de Lausanne (EPFL), Lausanne 1015, Switzerland; Biological and Environmental Sciences and Engineering Division, King Abdullah University of Science and Technology (KAUST), Thuwal 23955, Saudi Arabia; Blue Brain Project, École polytechnique fédérale de Lausanne (EPFL), Campus Biotech, Geneva 1202, Switzerland; Blue Brain Project, École polytechnique fédérale de Lausanne (EPFL), Campus Biotech, Geneva 1202, Switzerland

**Keywords:** 3D models, astrocyte, morphology, neuroanatomy, neuro-glia-vasculature, simulation

## Abstract

Astrocytes connect the vasculature to neurons mediating the supply of nutrients and biochemicals. They are involved in a growing number of physiological and pathophysiological processes that result from biophysical, physiological, and molecular interactions in this neuro-glia-vascular ensemble (NGV). The lack of a detailed cytoarchitecture severely restricts the understanding of how they support brain function. To address this problem, we used data from multiple sources to create a data-driven digital reconstruction of the NGV at micrometer anatomical resolution. We reconstructed 0.2 mm^3^ of the rat somatosensory cortex with 16 000 morphologically detailed neurons, 2500 protoplasmic astrocytes, and its microvasculature. The consistency of the reconstruction with a wide array of experimental measurements allows novel predictions of the NGV organization, allowing the anatomical reconstruction of overlapping astrocytic microdomains and the quantification of endfeet connecting each astrocyte to the vasculature, as well as the extent to which they cover the latter. Structural analysis showed that astrocytes optimize their positions to provide uniform vascular coverage for trophic support and signaling. However, this optimal organization rapidly declines as their density increases. The NGV digital reconstruction is a resource that will enable a better understanding of the anatomical principles and geometric constraints, which govern how astrocytes support brain function.

## Introduction

**Figure 1:**
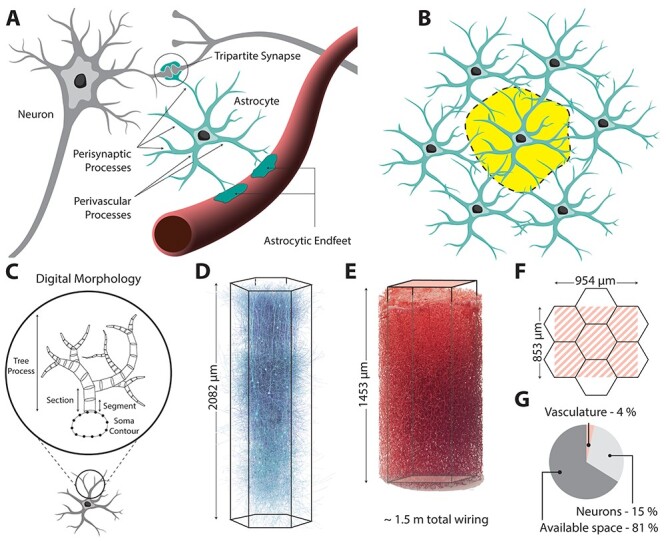
Neuro—glia—vascular architecture overview. (*A*) Astrocytes contact and wrap around synapses and project their perivascular processes to the surface of the vasculature, where they form endfeet. (*B*) Astrocytes establish anatomically exclusive domains, which minimally overlap with their astrocyte neighbors. (*C*) In silico digital morphologies are tree structures that connect to a central soma geometry. (*D*) In silico digital reconstruction of neuronal neocortical microcircuit of the somatosensory cortex. (*E*) Experimental reconstruction of cerebral microvasculature. (*F*) Overlap of neuronal mesocircuit and vasculature dataset. (*G*) Percentage of existing volume occupancy in the circuit space.

Neurons, the principal subjects of neuroscience, are structurally and functionally linked to glia and the microvasculature, forming a complex system of multidirectional communication known as the neuro-glia-vascular (NGV; Fig. 1*A*) ensemble (Verkhratsky and Toescu 2006). The most common type of glia, the protoplasmic astrocytes, radially extend between 5 and 10 primary processes (Damoiseaux and Greicius 2009; Di Benedetto et al. 2016; Calì et al. 2019; Moye et al. 2019), which ramify progressively into finer and finer branches, filling up their entire spatial extent (Bushong et al. 2002). Their fine leaflet-like processes wrap around the axon–spine interface (Ventura and Harris 1999; Genoud et al. 2006) forming tripartite units (Araque et al. 1999; Santello et al. 2012). They also extend one to five thick processes to capillaries, arterioles, and venules, attaching firmly on their surface (Kacem et al. 1998; Abbott et al. 2006; Mathiisen et al. 2010). Each astrocyte is in contact with 6–15 astrocyte neighbors (Xu et al. 2010), forming a network that is known as the astrocytic syncytium (Fig. 1*B*). Studying this system requires the combination of electrophysiological and high-resolution imaging techniques to monitor the NGV physiology, a technically challenging and expensive endeavor that has started to be tackled in recent decades (Zonta et al. 2003). An anatomically accurate model of all the structural components is needed to understand and investigate normal brain mechanisms and pathologies linked to the NGV.

Computational neuroscience has made remarkable progress since the creation of the first data-driven biological neuronal model in the 1950s by Hodgkin and Huxley (1952) and the subsequent introduction of multicompartment modeling of digitally reconstructed neuronal morphologies. A digital reconstruction of a cell morphology is a 3D trace, made either manually or using automated skeletonization techniques (Donohue and Ascoli 2011; Halavi et al. 2012). The branching morphology is represented as a sequence of segments (truncated cones) that capture the *X*, *Y*, *Z* coordinates, cross-sectional diameter, and connectivity links (Fig. 1*C*). Biologically realistic neuronal morphologies allow for compartmental biophysical models, which can capture the nonlinear dynamics arising from the distribution of ion channels throughout their compartments. The branching structure of neuronal morphologies is believed to play an important role in the computational tasks of the neuronal networks (Cuntz et al. 2007; van Elburg and van Ooyen 2010). By the same rationale, the branching topology and geometry of astrocytic morphologies are important for driving the propagation of calcium-induced waves that have been found to contribute to information processing (Cornell-Bell et al. 1990; Volterra and Meldolesi 2005; Hamilton and Attwell 2010; Araque et al. 2014), as well as biochemical compartmentalizations such as those involved in energy metabolism (Magistretti and Allaman 2018). However, because of the densely ramified nature of astrocytes, digital reconstructions using markers such as glial fibrillary acidic protein (GFAP) only capture the primary processes of the actual morphologies (Kulkarni et al. 2015). Three-dimensional electron microscopy on the other hand can provide the ultrastructure required for capturing the nanoscopic astrocytic processes but at the cost of low throughput (Calì et al. 2019).

In this work, we generated for the first time an in silico anatomical reconstruction of the P14 rat gray matter, composed of neurons, protoplasmic astrocytes, and the cerebral microvasculature at the level of biophysically detailed morphologies. To address the intricate structural organization of the NGV components, we combined prior work on the reconstruction of neuronal microcircuitry (Markram et al. 2015) with sparse literature data to develop a large-scale algorithmic framework for growing detailed astrocytic morphologies that connect to neurons, the vasculature, and other astrocytes while tiling the cortical space.

To achieve this, we first reconstructed the population-wide organization of the NGV. We colocalized neuronal and the vascular datasets (Fig. 1*E*–*G*) in a shared region, which was then populated with astrocytic somas. Next, we partitioned the cortical space into tiling and overlapping polygons, representing the microdomains of astrocytes. Within the boundary of each domain, the connectivity of the somata to synapses and to the vasculature was established.

To grow detailed astrocytic morphologies, we first solved the challenge of the small number of experimental astroglial morphologies, by extracting the branching topology of experimentally reconstructed astrocytes and using it to stochastically generate new morphologies (Kanari et al. 2018). We combined this approach with the population-wide data and geometrical constraints generated by the steps above into a novel synthesis algorithm that allowed to in silico grow unique astrocytic morphologies in their local environment. They formed connections with neuronal synapses, projected processes to the vasculature, and grew endfeet that wrapped around the surface of the vessels. We aimed to generate morphologically accurate astroglial cells in silico, rather than model their development.

Using the NGV digital reconstruction, we investigated the compositional and organizational principles that govern the underlying biological complexity. Specifically, we analyzed the colocalization of astrocytic somata, large vessels, capillaries, and endfeet targets on the vascular surface, unveiling the dominating elements of endfeet organization and what that may mean in terms of neuronal trophic support. We then generated multiple NGV realizations of increasing astrocyte densities and discovered a geometric limit in the ability of astrocytes to form endfeet and encapsulate synapses, which depends on the interplay of proliferation and contact spacing. Delving deeper into the NGV quantification, we found a systematic order-of-magnitude difference in the NGV composition that arises from purely geometric constraints and is observed in the literature. Finally, we showed it is possible to simultaneously quantify compositional (densities, wiring, surface areas, and volume) and organizational (connectivities, distance distributions, correlations) aspects of the NGV entities, opening a window to the prediction of numbers related to the NGV network organization.

A large-scale anatomical reconstruction of such a complex organization has not been possible before; however, it is an essential step towards understanding function in health and disease. Most importantly, we provide a data resource that can be used to investigate the morphological intricacies of the NGV anatomical reconstruction, and which paves the route for the simulation of physiology throughout an entire brain region, embedded in its actual cortical space with a biologically realistic spatial architecture, and offers the possibility to shed light on unknown questions about the microscopic brain interactions.

## Materials and Methods

A detailed description of the supplementary methods is available in the Supplementary Material S1.

### NGV Input Datasets

#### Neuronal Neocortical Mesocircuit

The neuronal component of the NGV circuit was digitally reconstructed using the circuit building framework presented in previous work by Markram et al. (2015). The neocortical mesocircuit consisted of a central microcircuit, occupying a volume of 0.28 mm^3^, with 23 590 neurons surrounded by six satellite microcircuits of 139 992 neurons in total. The overlapping arbors of neuronal morphologies formed ~8 million connections with ~37 million synapses, constituting the basis of the neuronal component of the NGV networks and providing the synaptic locations that are required from the NGV connectome.

#### Digital Microvascular Network Skeleton and Surface Meshing

A digital reconstruction of the rat’s cerebral microvasculature was produced by Reichold et al. (2009). Cylindrical blocks of the rat’s somatosensory cortex were scanned using synchrotron-based X-ray tomographic imaging at the TOMCAT beamline (Swiss Light Source). High-energy beams (20 KeV) irradiated the tissue with a resolution of 700 nm, resulting in grayscale image stacks, segmented into binary images and subsequently converted into midlines (skeleton) using custom software for artifact removal and skeletonization. A triangular discretization of the surface geometry was reconstructed using implicit structures known as metaobjects (Oeltze and Preim 2004; Abdellah et al. 2020), resulting in highly detailed meshes of the vascular skeleton (Supplementary Material S1).

#### Voxelized Brain Atlas Scaffold

Before the creation of an NGV circuit, a 3D atlas was first built. Its dimensions (954 × 1453 × 853 μm) were determined by colocalizing the vascular dataset (Reichold et al. 2009) with a neuronal mesocircuit (Markram et al. 2015) (Supplementary Material S1). The grid voxels (10 × 5 × 10 μm) were populated with astrocytic densities (astrocytes per mm^3^) extracted from the experimentally acquired profile corresponding to the somatosensory area of juvenile rats (Appaix et al. 2012). The voxelized atlas was used for placing astrocytic somas.

#### Experimentally Reconstructed Morphologies of Astrocytes

Image stacks of astrocytic morphologies from P14 rats were provided by the laboratory of Prof. Pierre Magistretti (King Abdullah University of Science and Technology). They were extracted using high-resolution serial block-face imaging, capturing the nanoscale structure of astrocytes with a 20 nm resolution (Calì et al. 2019). The stacks were converted into surface meshes, and the geometry was verified to be watertight and manifold. Finally, the surface meshes were contracted into skeletons consisting of nodes (coordinates and radii) and their connectivity (Shapira et al. 2008).

The reconstructed astrocyte morphologies were used both for the extraction of the branching topology of the P14 rat astrocytes (see Branching topology analysis) and for the calculation of morphometrics used in the topological synthesis and validation.

#### Topological Analysis of Experimental Astrocytes

In their work, Kanari et al. (2020) have shown that the distinct branching shapes of neuronal dendrites can be reproduced using the topological morphological descriptor (TMD) method (Kanari et al. 2018), which extracts a topological representation (persistence barcode) of a tree’s branching structure. A barcode is a collection of bars, with each bar encoding the start (root or bifurcation) and the end (termination) path lengths of a branch. The barcode is used to define the bifurcation and termination probabilities of each branch during the computational growth of morphologies. The coupling of these probabilities provides a method to implicitly reproduce key correlations between morphological features.

**Figure 2:**
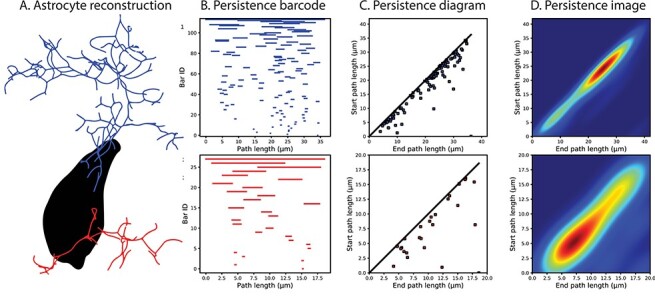
Example of the decomposition of astrocytic trees into persistence barcodes. (*A*) Two small trees of perivascular (blue) and perisynaptic (red) types were selected for demonstration purposes from an experimentally reconstructed astrocyte morphology (soma in black). (*B*) Each tree is decomposed into a barcode, with each branch being represented as a horizontal line (bar) marking the start and end path length from the soma. The barcode in (*B*) can also be represented as points in the persistence diagram (*C*), in which the start and end path lengths of each bar are shown as *y* and *x* coordinates, respectively. (*D*) By applying a kernel density estimator on the persistence diagram, the persistence image is generated, which shows the bar density of the branching structure.

In order to capture the branching topology of astrocytes, we applied the TMD method on experimentally reconstructed astrocytes. Because of their different structural role, perivascular and perisynaptic trees (Fig. 2*A*) were converted separately into two collections of barcodes (Fig. 2*B*). Each collection was then used in the synthesis of new astrocyte morphologies to reproduce their branching structure.

Persistence barcodes are alternatively represented as persistence diagrams, in which the start and end points correspond to the *y*- and *x*-axis of a 2D diagram (Fig. 2*C*). The diagonal on the persistence diagram corresponds to bars that have equal start and end path lengths, that is, zero length. Applying a kernel density estimator on the persistence diagram results in a persistence image (Fig. 2*D*), which shows the bar density as a 2D pixel image. The persistence image was used in topological validation because it allows calculating a topological distance between two images from the difference of the pairwise pixel values.

### Reconstruction Algorithms

#### Generating Astrocytic Positions

Astrocytes exhibit variation in both density (Appaix et al. 2012) and dispersion because of their tiling organization (Bushong et al. 2002, 2004). Previous placement algorithms placed cells reproducing their density (Markram et al. 2015; Erö et al. 2018) across layers and brain regions. However, astrocytic tiling results in a regular distancing between cells, which depends on animal species and age (Distler et al. 1991; López-Hidalgo et al. 2016).

**Figure 3:**
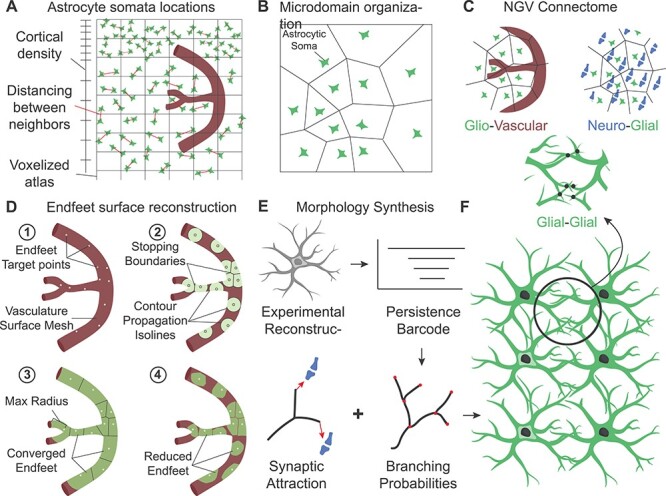
Reconstruction algorithms summary. (*A*) Cell placement. Astrocytic somata (green) were placed inside a voxelized atlas (grid), sampling from a spatial probability density that combined the cortical density with the distancing between astrocytes (red lines). (*B*) Astrocytic microdomains were generated from the location and size of astrocytic somas, modeled as a tessellation. (*C*) NGV connectome. The microdomains determined the accessible space for each astrocyte to establish connections with the vasculature (red) and the neuronal synapses (blue). Connectivity (black dots) between astrocytes (green) was determined after the morphologies have been grown, from the geometrical overlapping. (*D*) Growing of endfeet areas. Starting from the initial endfeet target points (*D*1), waves propagated following the geodesics of the vasculature surface, until they either reached an already occupied area or exceeded a maximum growing radius (*D*2). The converged areas were then truncated so that they match the input area distribution (*D*3–*D*4). (*E*) Morphology synthesis. Experimentally reconstructed astrocytic morphologies were converted into persistence barcodes, which determined the branching probabilities of in silico synthesized morphologies. The local directions of the processes was determined by the local attraction to synaptic terminals. (*F*) The synthesized morphologies were used to determine the connectivity between astrocytes and their neighbors, via geometric proximity.

In this work, the astrocytic locations were modeled as a random point pattern (Baddeley et al. 2007), the likelihood of which was governed by the Gibbs energy functional (Ruelle 1999; Dereudre 2019), combining the experimental densities registered in the atlas grid with pairwise repulsion to simulate a specific distancing between cells (Fig. 3*A*; Supplementary Material S1).

Astrocyte soma locations were sequentially sampled from the Gibbs density using the Metropolis–Hastings algorithm (Robert and Casella 1999). A collision of the newly generated soma with the vasculature geometry or already placed astrocytic somas would result in its rejection and the process would repeat until a trial soma is found that does not collide with other geometries.

#### Establishing Microdomain Organization

The anatomically exclusive territories of protoplasmic astrocytes were modeled as a partition of the 3D space into convex tiling polygons (Laguerre tessellation; Aurenhammer 1987). Their geometry was generated using the Laguerre distance, which combined the astrocytic soma positions and their radii (Supplementary Material S1). This geometrical abstraction generated bounding volumes, establishing the reachable region for each astrocyte (Fig. 3*B*). Regular tiling was converted to overlapping by uniformly scaling the domains until a 5% overlap was achieved.

#### Reconstructing the NGV Connectome

Three types of connectivities were reconstructed via the NGV circuit building pipeline (Fig. 3*C*): gliovascular connections (astrocyte–vasculature endfoot), neuroglial connections (astrocyte–neuron tripartite synapse), and glial connections (astrocyte–astrocyte gap junctions). The microdomains determined the available bounding region of each astrocyte. Therefore, the vasculature sites and the synapses each astrocyte can project to are limited to the inside of its microdomain geometry.

To establish the connectivity between astrocytes and the vasculature, we first distributed potential targets on the vasculature skeleton graph with a frequency of 0.17 μm^−1^ (McCaslin et al. 2011) and then determined which fraction of the resulting point cloud was included in each microdomain boundary polygon. Based on literature data, each astrocyte was assigned a number of endfeet, ranging from one to five (Moye et al. 2019), and the endfeet sites were sequentially selected according to the following observations and experimental astrocyte reconstructions: endfeet processes minimize their distance to the vascular site (Kacem et al. 1998), maximize the distance to nearby endfeet sites and target different branches (Calì et al. 2019).

Similarly, neuroglial connectivity was determined by first finding the synapses within each microdomain geometry. Then, a 60% random subset was selected to match the experimental observations (Reichenbach et al. 2010).

Finally, gap junctions between neighboring astrocytes were determined as touches between the colliding morphologies of the grown astrocytes, using the process of touch detection as presented in Markram et al. (2015). For this step, the full-grown astrocyte morphology was required (see Algorithmic growing of astrocytic morphologies).

#### Growing Endfeet Surfaces

The surface geometry of the endfeet wrapping around the vasculature was generated from the positions on the surface of the vasculature (endfeet target sites, Fig. 3*D*1), which have previously been assigned in the gliovascular connectivity step. From each endfoot target site, the endfeet area is grown isotropically across the vessel surface (Fig. 3*D*2) until it collides with another endfoot area or reaches a maximum radius (Fig. 3*D*3). The growth is considered competitive because all the endfeet are growing simultaneously restricting the area they are grown into from neighboring endfeet. After the simulation has converged, we pruned the overshoot surfaces (Fig. 3*D*3,*D*4) so that they match the experimental distribution of endfeet areas, which has a peak at 200 μm^2^ (Calì et al. 2019).

#### Algorithmic Generation of Astrocytic Morphologies

Astrocytes initially grow primary processes that radially extend outwards and become extensively ramified until they fill their tiling territories (Bushong et al. 2002, 2004; Ogata and Kosaka 2002). Within their domains, small branches exhibit increased coverage in proximity to synapses (Genoud et al. 2006), and it has been shown in a culture that astrocytes extend processes towards substances that are released by synapses (Hatten 1985; Cornell-Bell et al. 1990; Matsutani and Yamamoto 1997). Thus, to generate realistic astrocytic morphologies, three main challenges must be addressed: reproducing the complex branching patterns, creating many synthetic cells from a few experimental inputs, and embedding the cell growth in its local environment (colocalization, connectivity, tiling).

We developed a new algorithm that combined the topological synthesis of cells (Kanari et al. 2020) with the space colonization algorithm’s seed-driven growth (Runions et al. 2007), adapted for stochastically grown morphologies in the NGV circuit (Fig. 3*E*; Supplementary Material S1). Topological descriptors encoded the experimental branching patterns into hierarchical probabilities, which mandated when a growing astrocyte will branch or terminate. Decoupling the branching topology from the cell geometry allowed for the generation of a large number of astrocytes with uniquely grown processes in space. The growing tip direction in each morphology was influenced by its proximity to synapse locations, which served as attraction seeds. “Consuming” resources as morphologies grew guaranteed the exploration of the space by the movement of the growth front into areas that have available resources (Fig. 3*F*).

Due to a high surface-to-volume ratio in astroglial processes, it is difficult to capture their membrane geometry using a cylinder representation. For this reason, the volume and surface of segments were separately encoded in their diameters and perimeters. We first extracted morphometric distributions, such as the sibling ratio (ratio between diameters of daughter branches), diameter power relation (to model the relative diameters between parent and daughter branches), taper rates, trunk, and terminal diameters (Ascoli et al. 2008) from the experimentally reconstructed astrocytes. Then, the diameters were sampled for each astrocyte process, constrained by the aforementioned distributions (Kanari et al. 2020). For the distribution of perimeters, a linear regression model was fit on the diameter–perimeter pair values from all experimental reconstructions and used to predict the perimeters on the synthetic astrocytes from their distributed diameters.

#### Data Availability Statement

The data that support the findings of this study (experimental data, models and tools used in the circuit building process) are openly available in the NGV portal website at bbp.epfl.ch/ngv-portal. (NGV Portal 2021).

## Results

The available input vasculature dataset determined the bounding space of the generated NGV circuit, the dimensions of which are 955 × 1452 × 853 μm^3^, corresponding to a volume of 1.18 mm^3^, ~0.2% of the rat’s brain. A total of 14 402 astrocytes populated the bounding region, colocalized with 88 541 neurons and 1.37 m of vasculature wiring. The central microcircuit consisted of 15 888 neurons, 2469 astrocytes, and 0.23 m of the vasculature, occupying a volume of 0.2 mm^3^.

### Data Validation: Population Level

**Figure 4:**
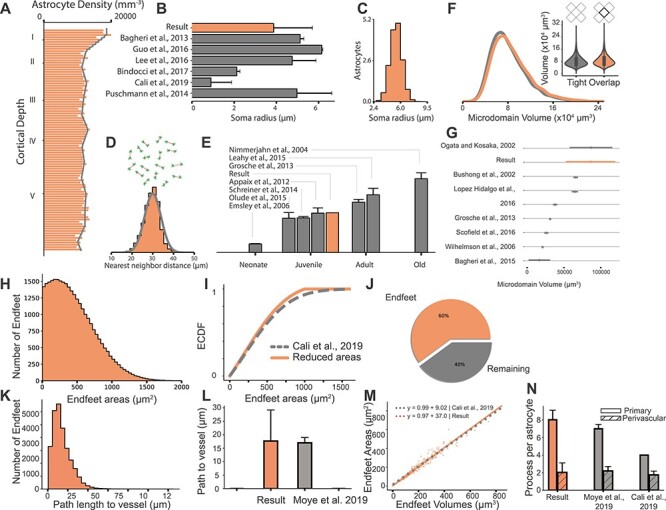
NGV data validation—population level. NGV circuit results are represented with orange, whereas literature data with gray, unless stated otherwise. (*A*) Astrocytic soma density comparison between the NGV circuit and reported values from Appaix et al. (2012). (*B*) Bar plot comparison between the circuit’s soma radius distribution and literature measurements. (*C*) The respective histogram of soma radius distribution. (*D*) Circuit’s histogram of the nearest neighbor distance distribution compared with the input constraint of López-Hidalgo et al. (2016) (gray line). (*E*) Average astrocyte density in context with measurements from various literature sources. (*F*) Comparison of volume distribution between tiling (gray) and overlapping (orange) microdomains. (*G*) Microdomain volume distribution comparison versus literature sources. (*H*) Pruned endfeet areas histogram and validation of its ECDF versus the target distribution (*I*) from Calì et al. (2019). (*J*) The surface area distribution results in a 60% coverage of the total vasculature surface area, leaving 40% uncovered (blue). (*K* and *L*) The shortest path length distribution from the soma to the vessel surface was validated against the measurements in Moye et al. (2019). (*M*) Reproduction of the endfeet volumes and areas relationship as measured in Calì et al. (2019). (*N*) Comparison of the number of primary and perivascular processes with respect to the literature.

To ensure biological fidelity, we validated that input constraints could be reproduced for each step in the circuit building process and compared structural measurements with corresponding values extracted from the literature and experimental data. Astrocytes were placed according to the densities reported in Appaix et al. (2012), measuring an average density of 12 241 mm^−3^, ranging from 9367 to 21 479 mm^−3^ close to the pia. The NGV framework accurately reproduced the density distribution for the selected profile (Fig. 4*A*), corresponding to P14 rat data. Other studies reported similar numbers (Supplementary Table S1 for a summary): 10 700 ± 1750 mm^−3^ in African giant rats (Olude et al. 2015) and 10 800 ± 400 mm^−3^ in mice (Schreiner et al. 2014). Astrocytic density increases from 2666 ± 133 mm^−3^ in neonates (Emsley and Macklis 2006) to 15 696 ± 860 mm^−3^ (Grosche et al. 2013) and 18 000 ± 1750 mm^−3^ (Leahy et al. 2013) in adults. Densities in old rodents do not exhibit a significant increase with reported values of 18 350 ± 1141 mm^−3^ (Nimmerjahn et al. 2004). Thus, the profile selection and generated densities lied within the range of juvenile numbers encountered in the literature (Fig. 4*E*).

Next, we validated the spatial association among astroglial somas to verify their orderly distribution. The distance between each astrocytic soma and its closest neighbor was calculated and the resulting distribution reproduced the target distribution from López-Hidalgo et al. (2016), with an average nearest-neighbor distance of 30 μm (Fig. 4*D*). During placement, the radii of the astrocytic somas were sampled from a normal distribution, which was fitted on the experimental values of astrocyte soma radii found in a multitude of studies (Bagheri et al. 2013; Puschmann et al. 2014; Bindocci et al. 2017; Calì et al. 2019) (Fig. 4*B*,*C*). However, due to the small size of the datasets, we allowed for higher variance instead of using any single one of these values to constrain our sampling method. The final result of the placement is astrocytic somas with their size linked to their positioning, introducing two improvements over previous methods of placing the somas as points and then assigning the geometry (Keller et al. 2018). Soma size is taken into account while being placed so that intersections of somas with other somas and the vasculature are eliminated.

The microdomain tessellation, representing the anatomically exclusive regions of the astrocytes, was generated from the position and size of the placed somas. The average microdomain volume was 81 725 μm^3^, ranging from 11 697 to 266 599 μm^3^, whereas the overlapping microdomains were measured at an average of 86 106 μm^3^, ranging from 12 324 to 280 890 μm^3^. The overlapping distribution exhibited a peak-to-peak shift of 2815 μm^3^ to the right, due to the higher domain volumes due to their overlap (Fig. 4*F*). In the literature, ages seem to be a determining factor in the size of the microdomains. Studies using adult animals report volumes from 16 400 to 31 000 μm^3^ (Wilhelmsson et al. 2006; Mishima and Hirase 2010; Bagheri et al. 2013; Grosche et al. 2013; Scofield et al. 2016). Juvenile rodent microdomain volumes have been observed to range between 65 900 and 86 700 μm^3^ (Bushong et al. 2002; Ogata and Kosaka 2002). The NGV model produced domain volumes that corresponded to juvenile astrocytic densities, capturing the magnitude from the respective studies (Fig. 4*G*). In the work of Lanjakornsiripan et al. (2018), specialized techniques were used to analyze astrocytic morphology, identifying a volume distribution ranging from 40 000 to 180 000 μm^3^. The most notable result of their quantification was that layer I astrocytes exhibited a smaller volume than in the rest of the layers (Supplementary Material S1). The NGV circuit reproduced this observation, which resulted from the significantly higher densities in layer I. Our quantification showed that the average size of the domains decreases as the average astrocyte density increases. This result provides an invaluable insight: the contact-spacing organization of astrocytes in biology induces constraints of purely geometric nature. This allows for the abstraction of astrocytes into mathematical entities, i.e., tessellation regions, verifying our initial assumption that astrocytic domains can be modeled as such.

The endfeet reconstruction simulation generated meshes that covered 91.1% of the vascular surface. Studies using chemical fixation for their tissues reported a 70%–100% coverage (Kacem et al. 1998; Simard et al. 2003; Mathiisen et al. 2010; Korogod et al. 2015) of the vasculature by perivascular endfeet. However, Korogod et al. (2015) showed that chemical fixation induces swelling of the astrocytic compartment, leading to increased coverage. They reported 62.9 ± 1.5% vascular coverage by astrocytic endfeet using anatomy-preserving tissue fixation. We reproduced a ~60% (Fig. 4*J*) vascular coverage by pruning the meshes using a Gaussian surface area distribution with a mean of 225 μm^2^ (Calì et al. 2019) and a standard deviation of 470 μm^2^. The latter was selected by optimizing the distribution for the reported coverage. The shortest path length from the soma to the vasculature’s surface was measured to be 16 μm on average (Fig. 4*K*), which was in agreement with reported values (Moye et al. 2019) (Fig. 4*L*). The endfoot size increased as the vessel diameter increased (slope = 4.6, *P* < 0.0001; Supplementary Material S1, section 2.1), a relationship that was also found by experimental measurements (Wang et al. 2020). Lastly, the relation between the surface area and thickness of the endfeet geometries validated that they agreed with the relationship from the study of Calì et al. (2019) (Fig. 4*M*).

In the NGV circuit, the number of perivascular processes was constrained for juvenile rodents at 2 ± 1 processes. These numbers are in accordance with literature measurements (Calì et al. 2019; Moye et al. 2019). Furthermore, the number of primary processes in the NGV was measured as 8 ± 1 processes (Fig. 4*N*). To validate the spatial relationship between neurons and astrocytes, for each astrocyte, the distance to the closest neuronal soma was calculated 13 ± 5 μm, ranging from 0.7 to 30 μm. The distribution falls within the range of literature observations, that is, from 5 to 30 μm for three types of inhibitory neurons (Refaeli et al. 2020).

### Data Validation: Astrocytic Morphologies

**Figure 5:**
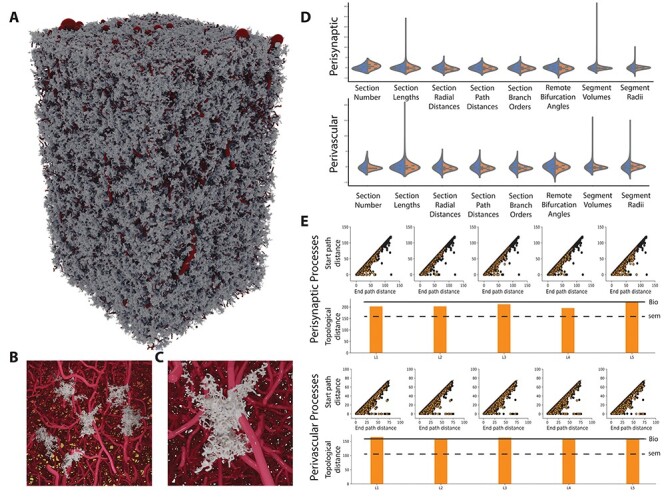
NGV data validation. (*A*) Entire circuit of synthesized astrocytic morphologies. (*B*) Example of six morphologies and a closeup (*C*) of a synthesized astrocyte and its respective endfoot on the vascular surface. (*D*) Feature comparison between synthesized (orange) and experimental (blue) astrocytes for perisynaptic and perivascular processes. (*E*) Per layer persistence diagrams overlap between the synthesized and experimental astrocytes. The topological distance of each layer’s persistence diagram compared with the experimental (black continuous line) and its respective standard error (black dashed line). Path distance units are in microns.

To validate the astrocytic component (Fig. 5*A*) of the NGV circuit, we randomly selected six nonboundary synthesized astrocyte representatives (Fig. 5*B*,*C*) from each layer. Morphometrics were extracted from these uniquely grown morphologies and were compared against those of the experimentally reconstructed astrocytes (Fig. 5*D*). The selection of morphometric features consisted of the number and length of sections, the section radial, path distances and branch orders, the remote bifurcation angles, and the segment radii and volumes.

**Figure 6:**
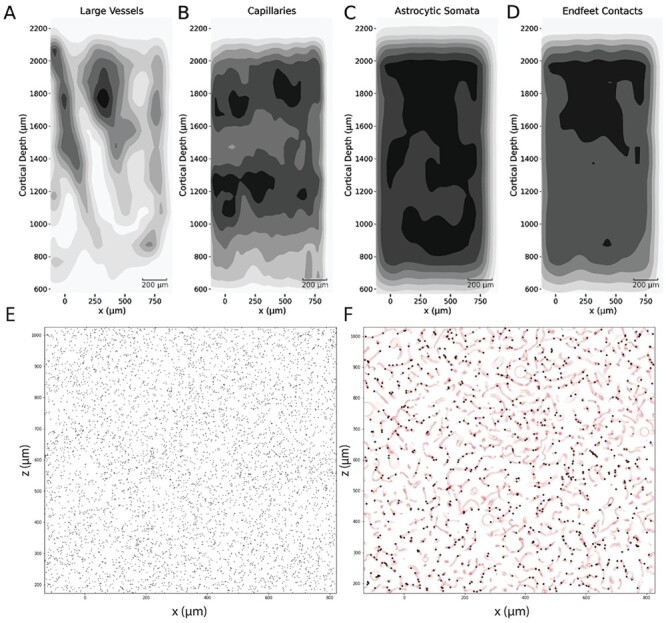
Spatial kernel density estimates plots of large vessels (*A*), capillaries (*B*), astrocytic somata coordinates (*C*), and endfeet targets on the surface of the vasculature (*D*). (*E*) Homogeneous distribution of endfeet targets in layer I. (*F*) A 30 μm slice in layer I of endfeet targets (black) and the vasculature surface mesh points (red).

The topological synthesis algorithm faithfully reproduced the population section length and path distance distributions, because it uses path distance from the soma as a metric for determining the branching locations. The radial distance distribution depends on the radial outgrowth of the synthesized trees, and although the experimentally reconstructed astrocytes exhibited varying sizes, the radial distances from all experimental morphologies were pooled together, smoothing out the distributions from longer and shorter cells. Therefore, when compared against a synthesized sample that drew from these barcodes randomly, similar statistics were achieved as some cells sampled smaller and others larger barcodes. Using a simple branching direction rule of one child following the parent direction and the second child following the synapse that is available and furthest away from the initial direction (Supplementary Material S1) was sufficient to reproduce the angle distributions of the experimental morphologies. This rule successfully encoded the behavior of astrocytes to create branches in a space-filling manner, as they progressively ramify during development (Bushong et al. 2002, 2004), filling up the available space. Therefore, using the synaptic cloud, which is already available from the neuronal circuit, the biological splitting behavior was reproduced without the need of extracting angle morphometrics from the experimental data, reducing in this way the input measurements that are required for astrocyte synthesis.

The trees of both reconstructed and synthesized morphologies were converted into persistence diagrams and subsequently into persistence images. The first step was to calculate the topological distance between each pair in the group of experimental morphologies in order to determine the error baseline within the reconstructed population. The average topological distance between reconstructed perivascular trees was 110 ± 32 units and for perisynaptic trees was 79 ± 24 units. Next, we separated the synthesized cells into five groups, one for each layer, and we calculated the topological distance between each pair of experimental and synthesized morphologies and averaged them for each group. For perivascular processes, in layer I, the average topological distance was 69 ± 27 units, in layer II, 68 ± 33 units, in layer III, 71 ± 40 units, in layer IV, 65 ± 33, and in layer V, 75 ± 33 units. For perisynaptic processes, in layer I, the topological distance was 54 ± 11 units, in layer II, 53 ± 9 units, in layer III, 54 ± 11 units, in layer IV, 52 ± 16 units, and in layer V, 53 ± 12 units (Fig. 5*E*). The comparison between the intra and per layer interpopulation topological distances showed that the reconstructed trees of the astrocytic morphologies exhibited large distances even when perisynaptic and perivascular processes were considered independently. One factor contributing to these results was the existence of trees in one morphology that were very short in radial extent as opposed to trees in a different morphology that were longer and spread out. Pruned trees could be the result of a partial reconstruction or cut from the reconstruction block. Given the small number of reconstructed morphologies (*n* = 3), further classification or curation was impossible. However, the synthesized trees exhibited smaller topological distances on average because of the sampling of the barcodes.

### Data Predictions

In experimental setups, there are a limited number of measurements that can be performed, depending on the protocols that were used to stain, fix the tissue, and digitally reconstruct it. In silico anatomical reconstructions of the NGV do not seek to replace these types of experiments but to minimize the costs and time required for scientific discoveries. Algorithmically generated NGV circuits can serve as magnifying glasses into the brain’s complexity, allowing scientists to explore the geometry and topology of its cells and their connections. Moreover, the creation of multiple NGV circuits, each one with a different set of parameters that reflect organizational changes in brain anatomy, will allow for a better understanding of the anatomical principles and their geometric constraints. All these insights can enable the scientist to construct more focused experiments. Here, we present an exploration of the quantification of some compositional and organizational aspects of the NGV circuit.

### Spatial Organization of Astrocytic Endfeet

To gain a general overview of the spatial organization of the gliovascular elements, spatial kernel density estimate plots were generated from the points comprising these datasets. A Gaussian kernel was used for the estimation of the probability density function, the bandwidth of which (standard deviation) was determined by Scott’s rule (Scott 2015). The plots were realized on the *x*–*y* plane, where *y* corresponded to the cortical depth of the circuit. The vasculature point samples were differentiated into large vessels (Fig. 6*A*) and capillaries (Fig. 6*B*) using a diameter threshold of 6 μm (Schmid et al. 2019). In addition, two more datasets were used for the density plots: the coordinates of the astrocytic somata (Fig. 6*C*) and the endfeet target points on the surface of the vasculature (Fig. 6*D*). The density plots showed no prominent spatial correlation between the endfeet targets and either the large vessels or the capillaries. They exhibited instead overlapping density regions with the density profile of the astrocytic somas, especially in layer I. In the NGV circuit, most astrocytes produce endfeet (>90% in the NGV circuit); therefore, the higher density of endfeet in regions of high soma density was a consistent prediction. As a matter of fact, the density of endfeet in the NGV circuit was measured as 23 464 endfeet per mm^3^ in layer V and 28 421 endfeet per mm^3^ in layer I. The cerebral microvasculature is a space-filling structure (Gould et al. 2011) that spans the entire cortex, while occupying <5% of the total cortical volume (Heinzer et al. 2006, 2008; Serduc et al. 2006). The astrocytic somata were evenly spaced following the density, which almost doubled in layer I. Therefore, it was found that the generation of the endfeet was not restricted by the vascular volume, and by extension most astrocytes always project endfeet to nearby vessels. Analyzing further the endfeet target point in layer I (Fig. 6*E*), we found that indeed the distribution of the endfeet targets was not “trapped” by the vascular structures, rather it was homogeneously distributed throughout the available space. Taking a closer look into a 30 μm slice verified that the endfeet target selection was spread out throughout the entire space, an observation that explained their spatial correlation with the astrocytic somas, but not with the vascular structures (Fig. 6*F*). In conclusion, the evenly spaced distribution of astrocytic somas throughout the neuropil allows for the generation of vascular endfeet projections, which extend to the vasculature from their local environment. The space-filling organization of the vasculature in combination with the astrocytic soma spacing allows for the uniform provision of glucose and nutrients to neurons (Magistretti and Pellerin 1996; Magistretti and Allaman 2018), which co-occupy the same space, and for an efficient recycling of water, neurotransmitters, waste molecules and ions (e.g., K+ clearance) (Abbott et al. 2010; Bellot-Saez et al. 2017).

### Effect of Astrocytic Density on Endfeet Organization and Synapse Encapsulation

Many studies suggest that astrocytic density varies little across brain structures and species when compared with the variation of the neuronal density due to differences in neuronal sizes (Tower and Young 1973; Haug 1987; Leuba and Garey 1989; Herculano-Houzel 2014). As neurons increase in size in bigger brains, their density decreases, leading to a higher glia/neuron ratio, ranging from 0.3 in rodents to 1.5 in humans (Nedergaard et al. 2003; Pelvig et al. 2008). However, recent studies show that astrocytes in humans span twice the width as their rodent counterparts (Oberheim et al. 2009), which raises the question of how morphological constraints in a dense neuropil affect the astrocytic population.

**Figure 7:**
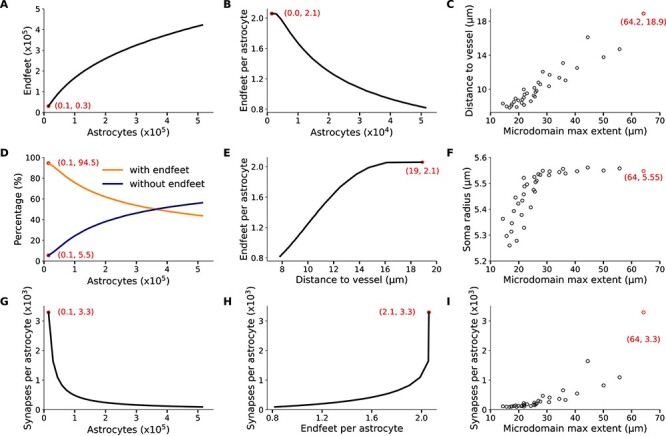
Effect of astrocytic proliferation on the feasibility of perivascular processes in the same bounding space. The red data points correspond to the reference circuit with the biological parameters. (*A*) Increasing the astrocytic density resulted in an increase in the endfeet numbers. (*B*) The number of endfeet per astrocyte decreased despite their total numbers. (*C*) The denser packing resulted in smaller distances to the vessels and domain extents. (*D*) Classifying the astrocytes into astrocytes with and without endfeet, we measured that as the number of astrocytes increased, astrocytes with no endfeet increased in number, (*E*) their distance to the closest vessel became smaller, and (*F*) because of the packing, there is a bias for smaller soma sizes. (*G*) The increase in astrocytic density within the same volume results in a lower percentage of encapsulated synapses compared with the reference circuit. (*H*) Relationship between endfeet and percentage of encapsulated synapses per astrocyte, which is a consequence of the shrinking of astrocytic domains (*I*) as density increases.

In order to explore the structural dynamics between astrocytic density and microdomains, we generated multiple circuit realizations equipped with astrocytic somas, microdomains, gliovascular, and neuroglial connectivities, starting from a total of 14 648 astrocytes and scaling up to 500 thousand, while keeping the bounding space, neuronal population, and vasculature wiring unchanged. We found that as the astrocytic density increased, leading to a higher total number of astrocytes, the number of endfeet did not increase accordingly, but follows a sublinear relation (Fig. 7*A*). This relation was a result of the reduction of the number of endfeet per astrocyte from an average of 2.1 to 0.8 (Fig. 7*B*), induced by the shrinking of the microdomain bounding space, the extent of which dropped from 64.2 μm down to 15 μm (Fig. 7*C*). A smaller microdomain extent reduces the reachable space of an astrocyte, which in turn results in the decrease of the number of astrocytes that have endfeet. Specifically, the percentage of astrocytes with no endfeet increased from 1.5% to almost 60% of the total astrocytes in the circuit (Fig. 7*D*) and because of the tight packing, the average distance of the perivascular astrocytes to the closest vessel dropped from 19 to 0.8 μm (Fig. 7*E*), with their somas essentially touching the surface of the vasculature and their anatomical domains occluding access to neighboring astrocytes. In fact, the packing becomes so dense that the sampling of the soma radii becomes skewed, thereby favoring smaller values because of the lack of available space (Fig. 7*F*).

We also investigated the effect of astrocytic proliferation on the synaptic encapsulation for each domain in the NGV circuits. Specifically, the number of encapsulated synapses was counted and an average value was calculated for each circuit realization. More astrocytes packed in the same bounding space resulted in a drop of synapses per astrocyte, from 3289 in the reference circuit to 93 in the highest density circuit (Fig. 7*G*). Astrocytes lost access to synapses at a faster rate compared with perivascular projections due to the dense spatial distribution of synapses and the sparse vascular wiring (Fig. 7*H*). Both loss of access to synaptic and vascular sites was a result of the shrinkage of astrocytic domains (Fig. 7*I*).

### NGV Compositional Hierarchy

**Figure 8:**
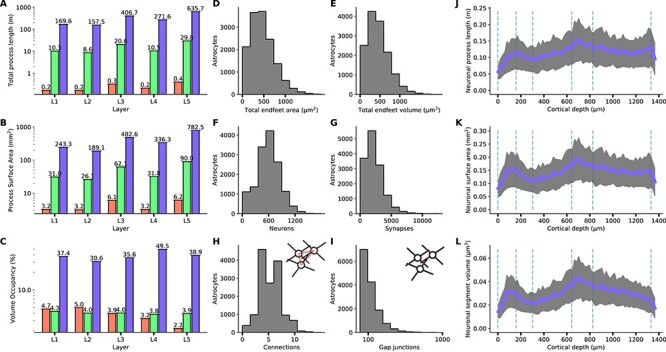
NGV data predictions. (*A*) Neuron (blue), astrocyte (green), and vasculature (red) total process length, surface areas (*B*), and volume fractions (*C*) per layer. (*D*, *E*) Total endfeet area and volume per astrocyte. (*F*, *G*) Number of neurons and synapses connected per astrocyte. (*H*, *I*) Number of neighbors and gap junctional connections per astrocyte. (*J*–*L*) Total neuron process length, surface area, and volume per astrocytic microdomain across the cortical depth.

In order to obtain a deeper understanding of the elements that comprise the gray matter, morphometric quantification was performed on the branches of neurons, astrocytes, and the vasculature in the NGV circuit. More specifically, the per layer total length, surface area, and volume were calculated from the morphology segments (Fig.8*A*–*C*), along with their respective densities (Supplementary Table S2). Neuronal processes occupied 33 ± 13%, astrocytes 4 ± 1%, and vasculature 3.8 ± 1.0% of the neuropil volume. It has been reported that neuronal dendrites occupy 35 ± 5% and axons 47 ± 5% of the neuropil volume (Mishchenko et al. 2010; Karbowski 2015). Astrocytes were reported to occupy 11 ± 4% of the neuropil volume (Mishchenko et al. 2010; Dienel and Rothman 2020), and vasculature <5% of the total cortical volume (Heinzer et al. 2006, 2008; Serduc et al. 2006). The missing volume percentage of the neuronal wiring in the NGV circuit corresponds to the missing afferent fibers from outside the region of interest, which have been predicted to be ~41 million and would have formed an additional 147 ± 4 million synapses (Markram et al. 2015). Astrocytic process volume fractions exhibited a 6% less coverage compared with experimental estimates, which could not be explained in terms of missing wiring due to their localized structure. However, the NGV circuit models the anatomical architecture of the P14 rat somatosensory cortex, in which the astrocyte density (12 286 ± 1601 mm^−1^) has not yet reached adult values (15 000 ± 18 000; Gordon et al. 2007; Leahy et al. 2013). In addition, the degree of astrocytic ramification increases significantly from P14 to P21, at which age it converges into the mature spongiform phenotype that covers the entire domain (Bushong et al. 2002, 2004). The synthesized morphologies of astrocytes in the NGV circuit were generated from the branching topologies of P14 reconstructed astrocytes (Calì et al. 2019), which have not yet acquired the mature phenotype. Therefore, under the light of these two predicates, i.e., lower soma density and ramification compared with mature astrocytes, the lower volume fractions in the NGV circuit were reasonable compared with the reported measurements on adult rodents.

Neuronal process total length ranged from 120 m in layer I to 657 m in layer V, two orders of magnitude higher than astrocytic process lengths, which ranged from 1.4 to 3.5 m in layer V and three orders of magnitude higher than vasculature wiring, which ranged from 0.2 m in layer I to 0.5 m in layer V. Total surface area for neurons ranged from 242 mm^2^ in layer I to 781 mm^2^ in layer V. Astrocytic process surface area was measured from 31 mm^2^ in layer I to 90 mm^2^ in layer V. Finally, vasculature surface area ranged from 3.2 mm^2^ in layer I to 6.2 mm^2^ in layer V. The ratio of the total length between neurons and astrocytes was 20 ± 3 and between neuronal and vascular total length was 1347 ± 372. The total process surface ratios were 8 ± 1 and 98 ± 29, respectively. Following the quantification of the geometrical features of neurons, astrocytes, and the vasculature, it was apparent that there was a systematic order-of-magnitude difference between them. The data suggested there is a hierarchy in cortical composition, the origin of which has been theorized in terms of length (Klyachko and Stevens 2003; Wen et al. 2009), conduction delay (Budd et al. 2010), volume or/and spine economy (Karbowski 2015) minimization. Most importantly, we showed here that an in silico circuit of the NGV architecture can indeed be used to investigate questions concerning the intricacies of cortical composition and their relation to computational capacity.

### Astrocyte-Related Numbers

The reconstruction can predict quantities that are difficult to access experimentally. We present here a set of measurements of the relation of astrocytes with neurons and the vasculature (Supplementary Table S3). Due to the fact that the gliovascular interface has been extensively analyzed in the validation of the NGV circuit, two additional quantities were extracted that were not found in the literature, namely the total endfeet surface areas and volumes for each astrocyte. The median of the total endfeet surface areas was 427 μm^2^ (Fig. 8*D*), and the median of the total endfeet volume per astrocyte was 414 μm^3^ (Fig. 8*E*). The neuroglial interface consists of the connections between neurons and astrocytes via the formation of tripartite synapses. Each astrocyte domain connected to 627 ± 259 neurons (Fig. 8*F*), whereas 6 ± 4 neuronal somata were in contact with each microdomain. The number of neuronal somata per astrocyte was in a consistent range compared with reported numbers of four to eight neuronal cell bodies per astrocytic domain in the rat hippocampus (Halassa et al. 2007). The median of the number of synapses per microdomain was 3010 (Fig. 8*G*), which was less than the 100 000 synapses per domain that have been reported by Bushong et al. (2002). This discrepancy is due to the missing afferent fibers, mostly long-range projections (Stepanyants et al. 2009). Taking into account the missing synapses from the external connections would result in a synapse density of 0.9 ± 0.1 μm^−3^ for layers I–V (Santuy et al. 2018), the predicted median for each microdomain would be 71 995 and the 95th percentile 134 010 synapses, which is consistent with the literature.

The astrocytic syncytium is formed via gap junctional connections, established in the overlapping interface between each astrocyte and its neighboring astrocytes. The synthesized astrocytes formed 5 ± 2 connections with their neighbors (Fig. 8*H*). Experimentally measured connections were reported as 11 ± 3 connections, ranging from 6 to 15 in hippocampal slices from P21 to P25 rats (Xu et al. 2010). In order to discern if the source of this difference in the emergent connections of the model was either a geometrical restriction resulting from the domain tessellation or morphological artifact, the number of neighbors per astrocyte was calculated based on the domain tessellation (using only the polygons). Therefore, analyzing the number of neighboring domains to each domain resulted in 15 ± 3 neighbors per astrocyte, which were notably higher than the connections established from the gap junctions. The connections were calculated from the detection of the intersections (touches) between neighboring morphologies. The median number of gap junctional connections was found to be 198, with the 95th percentile being 609 (Fig. 8*I*). The exponential distribution of the gap junction numbers in combination with the available neighbors signified that the NGV astrocytes, not being yet fully mature, did not exhibit extensive ramifications, allowing for a uniform interface across the boundaries of the domain. The primary processes reached the boundaries of the domain and penetrated into neighboring territory forming clusters of connections. This is indeed how astrocytes form connections with their neighbors while developing and before reaching the maturation stage (Bushong et al. 2002, 2004; Ogata and Kosaka 2002).

In silico circuits allow for continuous and simultaneous analysis of multiple features. For example, we estimated the average total wiring, surface area, and volume of neuronal branches per microdomain across the cortical depth (Fig. 8*J*–*L*). The total process wiring per microdomain ranged between 0.01 and 0.2 m, the total neuronal segment area per microdomains range between 0.05 and 0.2 mm^2^, and the total neuronal segment volume per microdomain ranged from 0.03 to 0.061 × 10^6^ μm^3^.

## Discussion

We have created the first data-driven digital reconstruction of the NGV structural organization at a micrometer anatomical resolution. Detailed network reconstructions are valuable resources for guiding insights on the emergent properties of their interconnected elements that give rise to brain complexity and function. However, a large-scale geometrical reconstruction that combines neurons, glia, and microvasculature has not been made yet. It was therefore the aim of this work to bridge this gap.

Using sparse data from numerous studies, we built a network of 15 888 neurons and 2469 protoplasmic astrocytes, forming a functional column of the P14 rat neocortex with its microvasculature. The state-of-the-art model of neuronal microcircuitry (Markram et al. 2015) was combined with an experimental reconstruction of the neocortical vasculature to algorithmically generate tiling astrocytic morphologies, which projected to the vasculature, formed endfeet, and connected to the neuronal synapses establishing tripartite ensembles. The topological generation of astrocytic morphologies was a paradigm shift from existing approaches (Savtchenko et al. 2018), limited by the number of experimentally reconstructed morphologies. Instead, by extracting the branching topology from scarce data, it was possible to generate unlimited morphologies, which replicated the input population’s biological branching topology, yet grown into unique, space-embedded, and context-aware forms. Their spatial co-occupancy was partially addressed on the somatic level by not allowing collisions of the somata with other geometric entities when placing them. Neuronal and astrocytic branches intersected because of the neuronal circuit’s composition of experimental, not synthesized, reconstructions. To apply intersection avoidance, strategies would require fully synthesized circuits (Torben-Nielsen and De Schutter 2014; Vanherpe et al. 2016) in the future, in which all cells would grow simultaneously.

To ensure biological fidelity, we validated the NGV circuit against numerous structural measurements with corresponding values extracted from the literature and experimental data in multiple levels of detail, ranging from somatic networks to single morphologies. Although extensively validated, the reconstruction certainly includes inaccuracies due to the combination of measurements from different studies with different experimental setups and an incomplete understanding of biological principles. Such inaccuracies will be resolved via the incremental integration of additional literature data and constraints as they become available. Finally, by providing the structural foundations for functional models of the blood–brain barrier (BBB) energetics and signaling, for calcium-induced waves throughout the astrocytic syncytium, modeling of blood flow, and for the establishment of tripartite synapses, it will be possible to further reduce inaccuracies via the validation thereof.

The NGV circuit was used to extract measurements at multiple levels of detail, recapitulating experimental findings and providing exploratory predictions of the underlying biological complexity. The spatial analysis of the astrocytic, vessel, and endfeet locations has shown that the endfeet locations did not form clustered regions parallel to the pia. Homogeneously distributed, the endfeet locations varied in density only across the cortical depth, reflecting the astrocytic density changes. The microvasculature does not influence the endfeet locations due to its space-filling geometry (Lorthois and Cassot 2010), allowing for a spatially continuous provision of trophic support to neurons throughout the cortical space as the majority of astrocytes can reach vessels in their vicinity. Therefore, the tiling astrocyte compartmentalizes the space-filling vasculature, and their endfeet optimize the communication wiring from the vascular site to neurons. Given the relatively low density of astrocytes compared with neurons, this endfeet organization and coverage would be impossible if astrocytes did not partition the cortical space with anatomically exclusive regions, and that would possibly lead to insufficient neuronal trophic support.

To further understand the implication of astrocytic density in endfeet organization and synapse encapsulation, we generated multiple circuit realizations of increasing astrocyte densities up to half a million astrocytes, with identical parameters and constraints. We found that as the total number of astrocytes increased, their overall extent shrank due to their tight packing, restricting their access to vascular sites. The higher astrocytic numbers did not compensate for the drop in endfeet appositions due to the tight packing of domains that prevented astrocytes from projecting to the vasculature. This shrinking effect due to contact spacing also resulted in a drastic drop in the number of synapses encapsulated by a single astrocytic domain. This would most likely disrupt the ability of astrocytes to integrate synaptic signals, resulting in altered spatiotemporal signaling. In contrast to neurons, astrocytic density varies little in different species and animal ages (Herculano-Houzel 2014). Thus, our experiments indicated that the contact spacing behavior, which gives rise to anatomically exclusive domains, acts as a global constraint for the astrocytes’ morphological steady state, which is reached at 1 month of age in rodents (Ogata and Kosaka 2002; Bushong et al. 2004). In addition, for the morphological domain to include the vascular sites within reach, a specific range of spacing is required, which depends on the intervessel distance. Therefore, the astrocyte’s role in providing trophic support and modulating synaptic signaling polarizes its morphology and constrains its location to maximize the connections from the vasculature to neurons.

Delving deeper into the NGV quantification, we extracted the per layer lengths, surface areas, and volumes, both in terms of total and density measurements. Compared with reported biological numbers, the volume fractions of neuronal processes were smaller than the values reported in the literature due to the missing afferent fibers that reach the circuit from outside. Also, the lower average density of the P14 rat neuropil combined with the partially ramified morphologies resulted in a volume occupancy that was 6% lower than reported values for adult animals. The data suggested there is a hierarchy in cortical composition, the origin of which has been theorized in terms of length (Klyachko and Stevens 2003; Wen et al. 2009), conduction delay (Budd et al. 2010), volume or/and spine economy (Karbowski 2015) minimization. Finally, we showed that an in silico circuit of the NGV architecture could indeed be used to simultaneously quantify both compositional (densities, wiring, surface areas, and volume) and organizational (connectivities, distance distributions, correlations) aspects of its entities, as well as to investigate questions concerning the intricacies of cortical composition and their relation to computational capacity. Additional biological details will be incorporated as more data become available, improving the integration of existing features while enforcing additional constraints on the model, increasing its biological fidelity. For instance, recent studies have identified new subtypes (Clavreul et al. 2019; Batiuk et al. 2020) of protoplasmic astrocytes, the morphological characteristics of which vary with cortical depths and regions. Additional reconstructions of these new subtypes and their respective spatial profiles are required to extract their topological characteristics and improve the current configuration. Future iterations of the NGV architecture will expand into deeper regions, including fibrous astrocytes (Matyash and Kettenmann 2010) and other types of macroglia, such as oligodendrocytes. In addition, the inclusion of microglia (Nimmerjahn et al. 2004; Davalos et al. 2005), which plays an important role in brain health and disease (Schafer et al. 2013), will introduce new computational challenges due to their constantly motile, dynamic nature.

The building process of NGV circuits is designed to be agnostic with respect to the species, age, and pathology. The region of interest, cell organization and rules, cell morphometrics, and branching topology constitute the set of input data that drives the building process without implicitly imposing parameters, rules, or hard-coded behavior. In this work, we provided as proof of concept a rectangular region for a specific species and age, the atlas of which was manually constructed. Future extensions will allow users to select a region (or the entire brain) from existing atlases and build the local architecture directly inside the atlas frame of reference. For each new region, a new set of input data will be needed, which can be obtained in a similar manner as shown in this work.

Biophysical models of the BBB interface and its metabolic signaling require precise geometrical specifications of the astrocytic endfeet. Therefore, the algorithmic reconstruction of the endfoot-apposing surface allows for the modeling of the functional interface between astrocytes, pericytes, smooth muscle, and endothelial cells. The algorithmic approach of the present study allows for the reconstruction of all endfoot surfaces in a circuit, across the entire microvasculature in the region of interest. Changes in the endfeet surface areas, which lead by extension to the variation in the coverage of the vasculature, have been observed in pathologies such as major depressive disorder (Rajkowska et al. 2013) and Huntington’s disease (Hsiao et al. 2015). For example, the extent of the endfoot area determines total counts of the Kir and BK potassium channels that need to be distributed for a model of potassium buffering in the NGV unit (Witthoft et al. 2013). Therefore, a valid distribution of abutting endfeet areas is an integral part of a functional model of the BBB. A crucial factor for the accuracy of the endfeet areas is the quality of the vascular surface mesh. Disconnected components, floating segments, and reconstruction artifacts will all negatively influence the faithful reconstruction of the endfeet, as they will either trap the growth of an endfoot surface or force it to grow on a nonexistent structure. In our model, vasculature reconstruction errors are present and influence the endfeet area distribution; however, with upcoming high-quality datasets, such sources of error will be reduced.

The structural anatomy of the NGV system has consequences upon pathologies and concomitant treatment. Drug delivery research studies the molecular properties of drugs, but should also take into account the interaction of the drug with its environment, that is, the transit of the drug through the BBB to various locations of a healthy and/or pathological brain. Similarly, research in neurodegenerative diseases such as Alzheimer’s disease target reactive astrocytes, the morphology of which is entirely transformed with variation in their ramification, overlap, and proliferation compared with healthy brains.

Our model provides the structural foundation for the large-scale biophysical modeling of cross-talk between neurons, glia, and vasculature. This data-driven approach allows for incremental refinement as more experimental data become available, new biophysical models get published, and new questions arise.

## Supplementary Material

supplementary_material_bhab254

## Notes

The authors thank Jay Coggan for the helpful conversations and his editing contributions throughout the manuscript. The authors also thank Caitlin Monney for designing the table of contents illustration and cover artwork, Karin Holm for editing, Claudia Savoia for project coordination, Jean-Denis Courcol, Pavlo Getta, and Stefano Antonel for technical support, and Bruno Weber for helpful discussion and access to previously-published data. Lastly, the authors thank Nicolas Antille for his insights, visuals, and feedback during the early stages of this work. *Conflict of Interest*: None declared.
