# Supplementary material for: Digital Reconstruction of the Neuro-Glia-Vascular Architecture
Source: Cereb Cortex. 2021 Aug 13;31(12):5686–703. doi: 10.1093/cercor/bhab254 (PMC8568010; doi:10.1093/cercor/bhab254)
Supplement: supplementary_material_bhab254 [file supplementary_material_bhab254.zip › supplementary_material_bhab254.pdf]

Digital reconstruction of the neuro-glia-vascular architecture Supplementary Material

Eleftherios Zisis Daniel Keller Lida Kanari
Alexis Arnaudon Michael Gevaert Thomas Delemontex
Benoît Coste, Alessandro Foni Marwan Abdellah
Corrado Calì Kathryn Hess Pierre Julius Magistretti
Felix Schürmann Henry Markram

Contents

1 Supplementary Methods **2**
1.1 Programming environment and NGV framework 3
1.2 Voxelized brain atlas scaffold 5
1.3 Digital microvascular network skeleton and surface meshing . . 7
1.4 Generating astrocytic positions 9
1.5 Connectivities 12
1.6 Topological synthesis 15
1.7 Branching and termination 22
1.8 Vasculature attraction field analysis 25
1.9 Surface area and volume distribution 27
1.10 Endfeet surface reconstruction & pruning 29

2 Supplementary Figures **32**
2.1 Endfeet morphometrics 33

3 Supplementary Tables **35**

1 Supplementary Methods

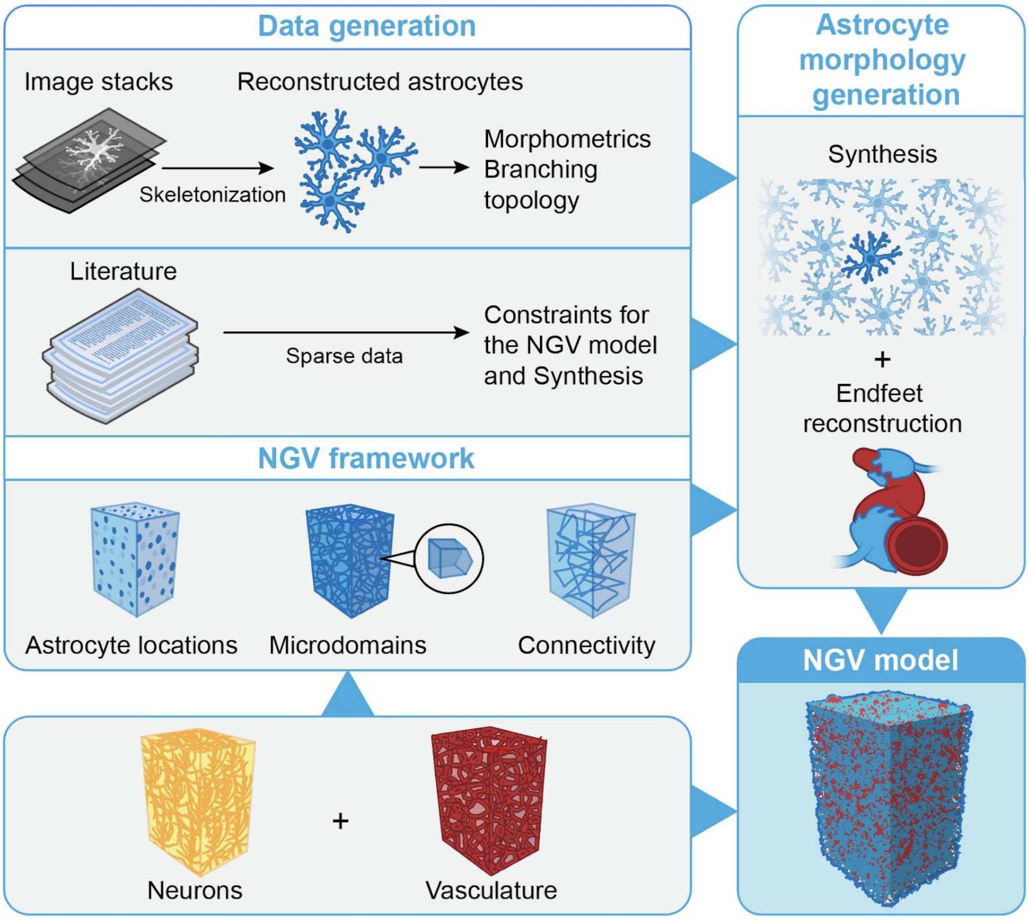

Figure 1 NGV framework overview

1.1 Programming environment and NGV framework

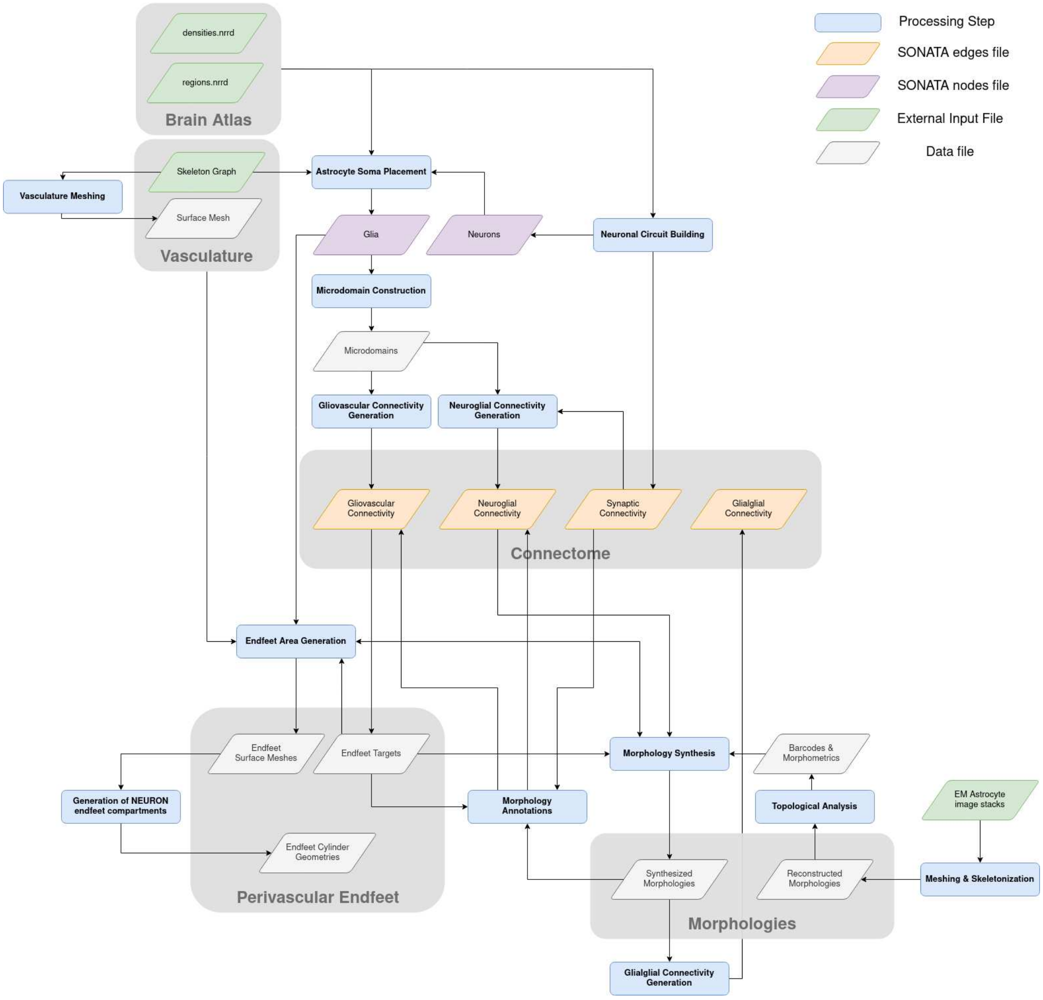

Figure 2 The main input datasets to the ngv frameworks are the brain atlas intensity and region of interest, the vasculature skeleton and surface mesh, the neuronal population from the neuronal circuit building pipeline and the astrocyte reconstructions required for synthesis. Each step also requires parameters for its algorithms that comprise the parameter profile. The data outputs from the framework are split into 3 categories: node populations and their properties (e.g. microdomains for astrocytes), edge populations or connectivities and their properties (e.g. endfeet areas for gliovascular connectivity) and synthesized morphologies.

The NGV circuit building framework was written in Python3 following a component-based design. A component is defined as a standalone processing step with strictly-defined inputs and outputs. The execution of a component may depend on the output files of more than one other component. This relationship between components results in an acyclic graph of dependencies

(DAG), which is managed by the Snakemake workflow engine (Köster and Rahmann, 2012). There are a total of thirteen processing steps as shown in Figure 7, and three main data categories in the framework: external inputs, cell-related data, connectivity-related data. External inputs are separated into input datasets and configuration parameters. Input datasets, such as the region of interest, astrocytic densities, the vasculature, etc., represent all the data that is used by the NGV framework. Configuration parameters include algorithm constants and NGV profile density distributions, which configures how the framework consumes the external datasets and how the algorithms are set. Different species, ages, and pathologies each have distinct profiles that need to be parameterized first in order to generate a representative NGV circuit. NGV cells and connectivities are stored in the SONATA network specification (Dai et al., 2020), as nodes and edges in HDF5 binary files. Blue Brain's libSONATA library provides support for the reading and writing of HDF5 node and edge datasets.

Each network entity collection, such as neurons and glia, is stored as a node population. Each cell in the node population is identified by an identifier (index). Node populations include attributes related to their spatial embedding (soma position, soma radius, orientation, layer, etc.), their morpho-electrical characteristics (morphological type, electrical type, etc.), and other information which emerges from the circuit building, such as the morphology filepath. Astrocytes, which are stored as glia with an ASTROCYTE morphological type, map to an additional dataset: the microdomains.

Connectivity between two node populations is registered as an edge population. The NGV architecture is established by four edge populations: synaptic, neuroglial, gliovascular, and glial-glial. Synaptic connectivity was produced during the neuronal circuit building pipeline and is an input for the NGV building along with the neuronal node population. Neuroglial, gliovascular and glial-glial connectivities represent the connections between neurons and astrocytes, astrocytes and the vasculature and astrocytes and their neighbors respectively. The gliovascular edge population also stores an endfoot identifier, which provides access to the endfeetome data structure containing the mesh of the endfeet that have been grown on the surface of the vasculature.

1.2 Voxelized brain atlas scaffold

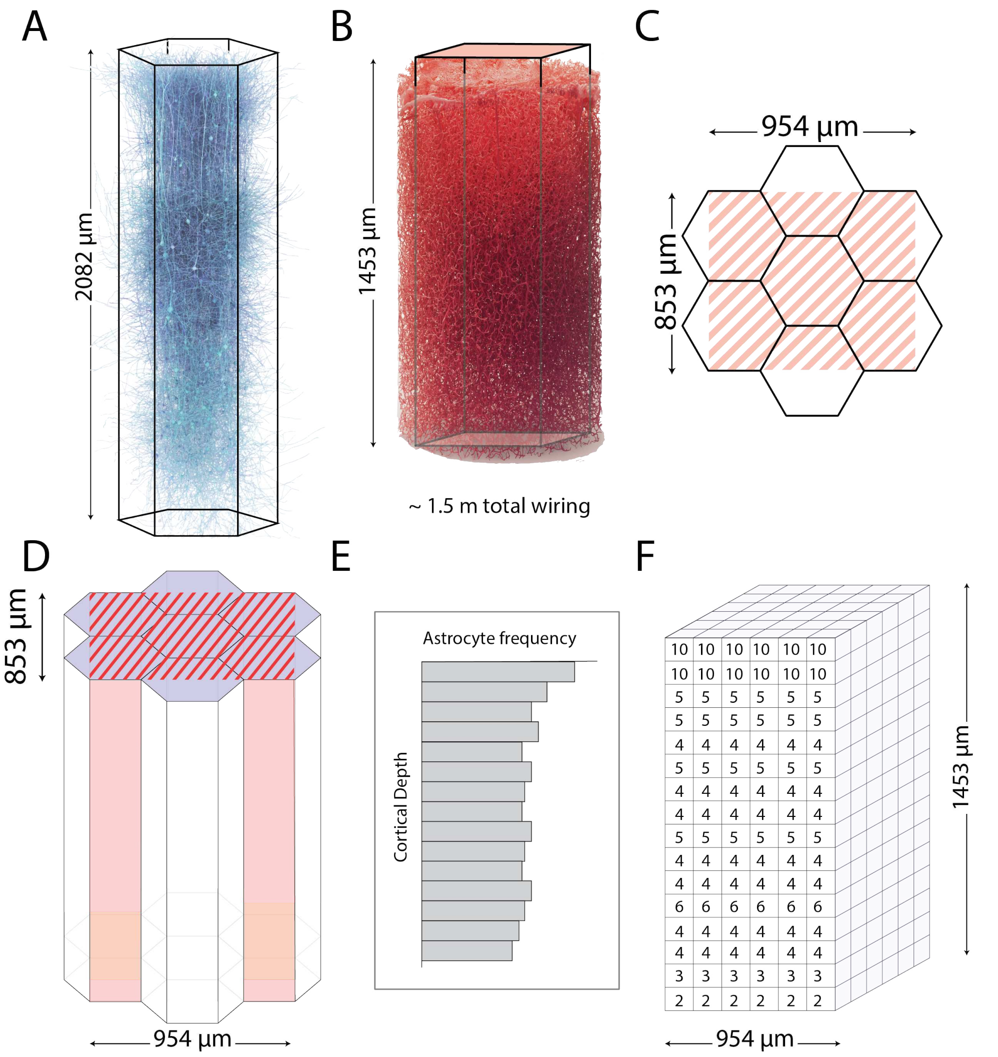

Figure 3 The creation of a brain atlas virtual circuit for the NGV requires the co-localization of seven microcircuits (A) and the microvascular dataset (B). The bounding box for the NGV circuit is the co-occupied space for both neuronal and vascular datasets (C,D). (E) 1D astrocyte frequency profile. (F) Rectangular grid with astrocyte counts, interpolated from the frequency profile

An atlas is a voxelized region equipped with circuit building essential information, such as brain region annotations, densities, cell types etc. (Erö et al., 2018). To create an NGV circuit, the extend of its spatial embedding, i.e. its bounding region, must be determined. We manually constructed a virtual 3D atlas for the NGV, ensuring the alignment of the neuronal and vascular coordinate systems. The microvasculature dataset was defined in a coordi-

nate system where the z coordinate corresponded to the cortical depth of 1453 µm. The neuronal mesocircuit (Markram et al., 2015), which was composed of seven microcircuits (Fig. 3A) hexagonally tiled, extended down to deep cortical layer 6 at 2082 µm across the y axis. Performing translation and rotation transformations we aligned the vasculature dataset to match the neuronal coordinate system, ensuring overlapping medial axes across y (Fig. 3C) and aligned the tops of both datasets at the pia. Due to the difference in height (vascular 1453 µm vs. neuronal 2082 µm), layer 6 was omitted from the NGV circuits, because there was no vasculature wiring covering that region. The spatial intersection of the aligned datasets (Fig. 3D) resulted to a bounding region of 954 µm $\times$ 853 µm $\times$ 1453 µm, which was used throughout the framework.

The voxelized atlas was constructed as a grid spanning the bounding region, in which the voxel y dimension (depth) was chosen to reflect the discretization resolution of the input frequency profile, which was 5 µm. A cell frequency profile is an 1D histogram of the number of astrocytes per mm^3 binned across the cortical depth (Appaix et al., 2012). Not having bin dimensions available for the lateral dimensions, x and z voxel size was arbitrarily selected to be 10 µm. The astrocytic frequencies were mapped to the 3D voxelized grid, by assigning a constant frequency on the xz plane and the profile values to the voxels in the y-direction (Fig. 3F).

1.3 Digital microvascular network skeleton and surface meshing

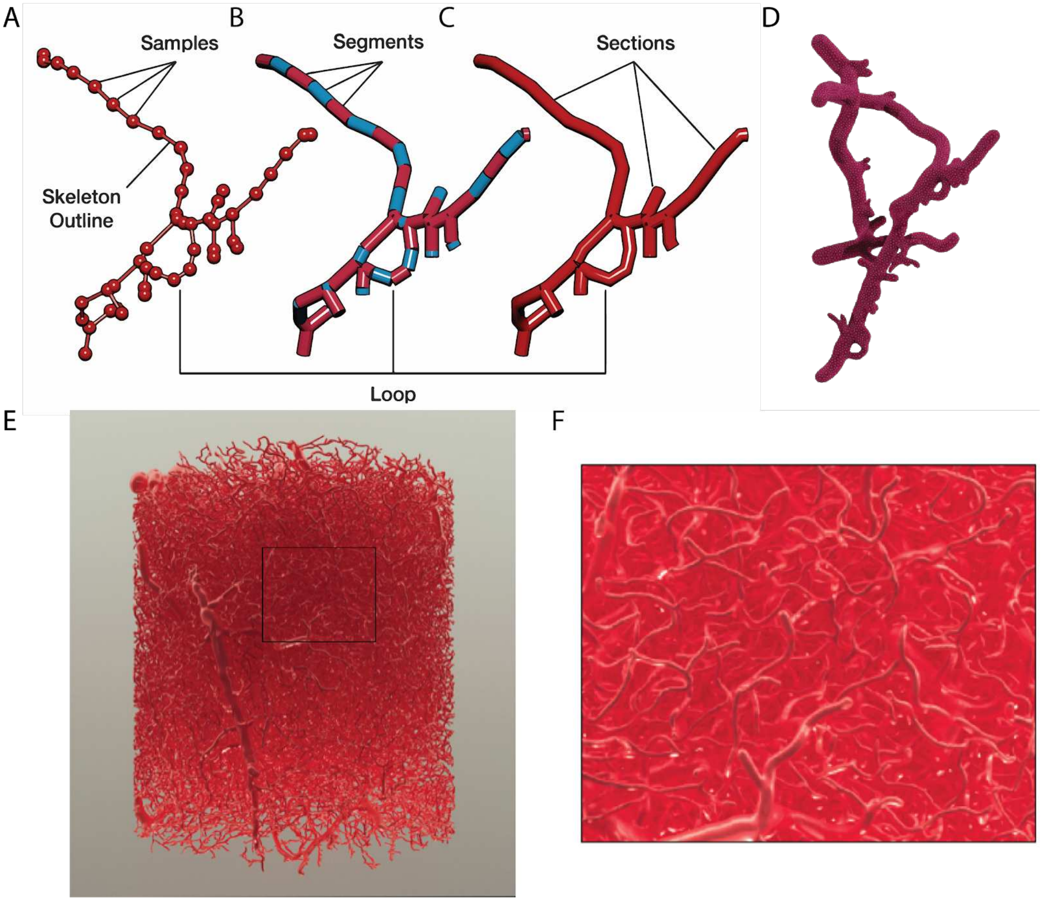

Figure 4 Vasculature meshing (A) The vasculature dataset consists of points with a diameter linked by edges. (B) Two consecutive points linked by an edge are defined as a segment. (C) A chain of consecutive points between two forking points is defined as a section. (D) Generated surface mesh with overlaid triangles. (E-F) Original vasculature dataset mesh.

A digital reconstruction of a rat cerebral vasculature dataset was produced by Reichold et al. (2009). Cylindrical blocks of the rat's somatosensory cortex vasculature were scanned using synchrotron-based X-ray tomographic imaging at the TOMCAT beamline (Swiss Light Source). High energy beams (20KeV) irradiated the tissue with a resolution of (700 nm), resulting in grayscale image stacks, which were segmented into binary images and subsequently converted into midlines (skeleton) using custom software for artifact removal and skeletonization.

The dataset consists of point samples linked together with edges (Fig. 4A). Each point is acquired with a diameter which represents the width of the cross section. Two consecutive points constitute a segment in the morphology (Fig. 4B), and consecutive segments form a morphological section (Fig. 4B). There are two representations of the skeleton dataset that are encountered: The point representation, in which a node is a single point in the morphology and an edge links two points, and the section representation, in which an entire section is clustered into a node and the edges linking sections together. Depending on the nature of the algorithm one or the other representation may be favorable for efficiency or memory consumption. Geometric discontinuities due to the reconstruction process that resulted in small gaps in the skeleton were connected based on the closest distance between components.

In the NGV framework, I distinguish between two types of datasets: the vasculature skeleton and the surface mesh. Although the majority of the steps in the circuit building pipeline used the skeleton of the cerebral microvasculature, the endfeet surface reconstruction required a more detailed representation of the surface geometry. Thus, starting from the skeletonized dataset we generated a triangular discretization of the surface geometry with variable resolution. The surface mesh is generated based on implicit structures (Fig. 4D), known as meta-objects (Oeltze and Preim, 2004), allowing for the creation of highly-detailed meshes of vasculature datasets (Fig. 4E,F) (Abdellah et al., 2020).

1.4 Generating astrocytic positions

An unordered set of positions (point pattern) $\mathbf{p} = \{p_1, p_2, ..., p_n\}$ can be modelled as a realisation of the Gibbs spatial point process $\mathbf{P}$ with a general probability density function (Dereudre, 2019; Ruelle, 1999):

$$f(\mathbf{p}) = \frac{1}{Z} h(\mathbf{p}) \qquad\qquad h(\mathbf{p}) = exp\left(-U(\mathbf{p})\right) \tag{1}$$

where $h(\mathbf{p})$ is the unnormalized density, U denotes the energy or potential of the point set interactions, and Z is a normalizing constant. Astrocytic somata in the neuropil exhibit a non-uniform laminar organization which can be modeled via a first order intensity $\lambda(p_i)$, which expresses the number of somata per unit of volume. Their contact spacing behavior on the other hand can be modeled as a second order pairwise repulsion interaction $r(\|p_i - p_j\|)$ for every $i \neq j$. Thus,

$$U(\mathbf{p}) = \sum_{i=1}^{n} \left(-\lambda(p_i) + \sum_{j \neq i} r(\|p_i - p_j\|) \right) \tag{2}$$

In order to simplify the combinatorial nature of the pairwise interaction we will instead switch to a repulsive influence exclusively from the nearest neighbor. Furthermore, a simple $1/r$ repulsion will be used:

$$\sum_{j \neq i} r\left(\|p_i - p_j\|\right) \sim r\left(\min_{j \neq i} \|p_i - p_j\|\right) \qquad\qquad r(d) = \frac{r_0}{d} \tag{3}$$

assembling all the above the unnormalized Gibbs density of the spatial point process for astrocytic somata point patterns has the form:

$$h_g(\mathbf{p}) = exp \sum_{i=1}^{n} \left(\lambda(p_i) - \frac{r_0}{\min_{j \neq i} \|p_i - p_j\|} \right) \tag{4}$$

We can now add further constraints on the soma generation by creating hybrid unnormalized densities. A constraint can be thought as a conditioning event $\mathcal{A}$ which is expressed by the indicator function $h_i = \mathbf{1}\{\mathbf{p} \in \mathcal{A}\}$. Conditioning events include out of the bounding box restriction and overlapping

with other geometrical elements, such as astrocytic or neuronal somata and the vasculature.

The resulting hybrid unnormalized density:

$$h(\mathbf{p}) = h_g(\mathbf{p}) \prod_{i=1}^{k} h_i(\mathbf{p}) \tag{5}$$

For the overlap is not sufficient to check if the placement of a new point collides with another geometric entity. An astrocytic soma has volume, thus we can interpret it as a sphere of a certain radius. When a placement trial is taking place, the radius is sampled from a truncated normal biological distribution and then the check for the overlap is performed.

In order to simulate the generation of a point pattern we need to form the Papangelou conditional intensity, which is the ratio of the density function with a new position u and without it:

$$\lambda_p(u, \mathbf{p}) = \frac{f\left(\mathbf{p} \cup \{u\}\right)}{f\left(\mathbf{p}\right)} = \frac{h\left(\mathbf{p} \cup \{u\}\right)}{h\left(\mathbf{p}\right)} \tag{6}$$

It is evident that the normalizing constant is not included in this formulation.

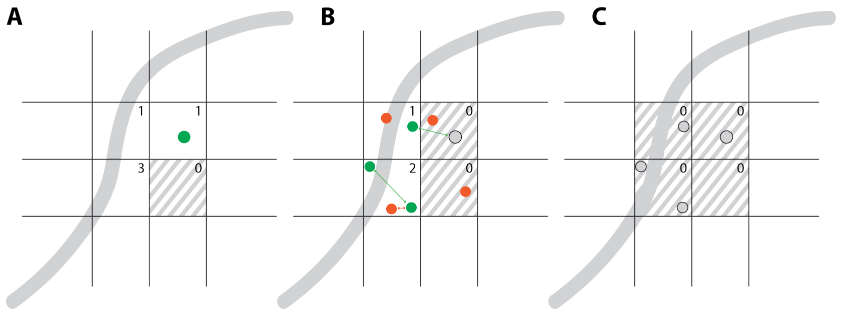

Figure 5 Placement steps: Green circles represent valid soma placement trials, red the invalid and gray the accepted ones from the previous step. Similarly, green and red lines represent valid and invalid nearest distance evaluations respectively. The continuous gray line represents the geometry of a vessel, which crosses the grid and influences placement. (A) The astrocytic density is converted into astrocyte counts, which are reduced when a soma is placed into the respective voxel group. (B) A placement trial is rejected if it takes place into a maxed-out voxel, or if it collides with other geometrical entities. (C) Placement finishes when either all the voxels have been filled or there is no available space to put more somata.

The placement algorithm starts by clustering the voxels intro groups G_i of equal intensity d_i and the total count of the cells in each group N_{G_i} is estimated:

$$N_{G_i} = N_{v \in G_i} \times V_{\text{voxel}} \times d_i \tag{7}$$

Each time a soma is placed the respective voxel count is reduced by one (Fig. 5A-B). For each placement trial, a position is chosen uniformly from the non-occupied voxels and a radius is sampled from a normal distribution with mean $\mu = 5.6$ and standard deviation $\sigma = 0.7$, extracted from literature data. If there is no collision of the trial sphere with the vasculature skeleton, neuronal somata or already placed astrocytic somata, the energy functional is evaluated and the trial is accepted or rejected according to the Metropolis-Hastings algorithm. If not accepted, a new position / radius combination is generated and the procedure repeats until all voxels have reached their total numbers. The algorithm finishes when all cell counts have been filled or there is no available space left (Fig. 5C).

1.5 Connectivities

Three types of connectivities are reconstructed via the NGV circuit building pipeline: gliovascular connections (astrocyte-vasculature endfoot), the neuroglial connections (astrocyte-neuron tripartite synapse) and glial connections (astrocyte-astrocyte gap junctions). The generated microdomains were used to specify the accessible space for each astrocyte, allowing queries for the geometrical elements withing their boundaries. To optimize the spatial search queries of the point clouds, R-Tree (Beckmann et al., 1990) spatial data structures were built for the neuronal synapses and the vasculature skeleton.

Gliovascular

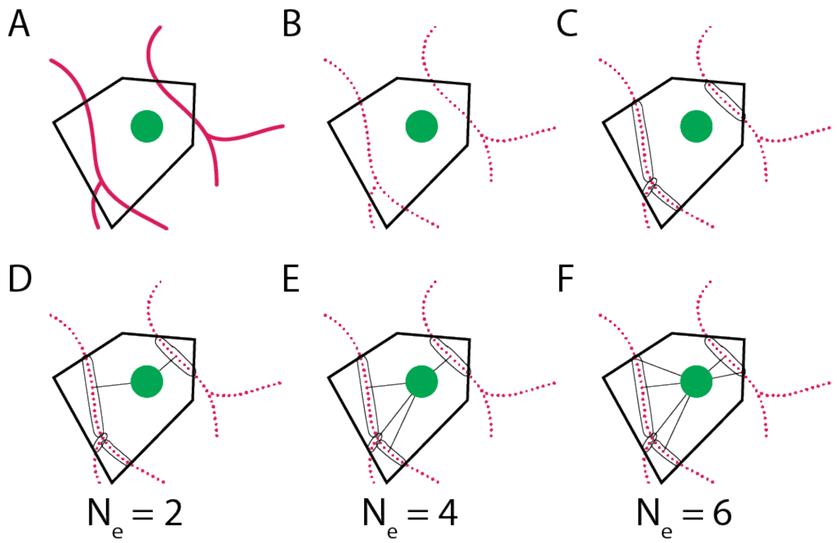

Figure 6 Algorithmic overview of gliovascular connectivity. (A-B) Potential targets are distributed on the vascular skeleton. (B-C) The targets included in the astrocyte microdomain are grouped by their section id. (D-E) If the number of endfeet is smaller that the number of components, the closest vertex from the closest components is assigned to them. (F) Otherwise, after assigning the closest vertex from each component, the algorithm iterates over each group finding a vertex that maximizes its distance to the closest selected target.

A set of points $P = \{ p_1, p_2, ..., p_n \}$ was distributed on the skeleton of the vasculature (Fig. 6A,B), according to a linear frequency of $0.17\,\mu m^{-1}$ (McCaslin et al., 2011), representing potential endfeet locations. For each astrocyte, a spatial query was performed using the microdomain boundary to find the potential points included in the respective domain. The selection of the actual endfeet targets from the potential point cloud of endfeet locations is

realised via the reachout algorithm, which draws a number for the endfeet number $N_e \sim \mathcal{N}(\mu_e, \sigma_e^2)$, where (μ_e, σ_e) are input parameters. The reachout algorithm first generated N_G groups, clustering the points by the vasculature section (for vasculature morphology definitions see section 1.3) they belong to , creating one group per section (Fig. 6C). The groups are then sorted by distance of the soma x_s to the closest point in each group. If $N_e \leq N_g$, then the closest point in the closest components are assigned as endfeet targets (Fig. 6D). Otherwise, the points are selected one at a time from each group, starting from the closest point of the closest group. When one point has been selected from each group, the algorithm iterates again over the groups selecting the points that maximize the distance to the already selected nearest neighbor (Fig. 6E,F). In the end, from the lines that connect the soma to the selected endfeet target locations, the intersection with the surface of the vasculature is calculated, generating the respective endfoot target on the surface of the vasculature.

Based on literature data, each astrocyte was assigned a number of endfeet, ranging from 1 to 5 (Moye et al., 2019). The optimization choices of the reachout algorithm were based on observations on experimental astrocyte reconstructions: endfeet processes minimize their distance to the vascular site (Kacem et al., 1998), maximize the distance to nearby endfeet sites and target different branches (Calì et al., 2019).

Neuroglial

The centroids of the synaptic connections were calculated by averaging the presynaptic and postsynaptic terminal locations for each synapse present in the neuronal circuit (Markram et al., 2015) (Fig. 7A). For each astrocyte, a spatial query was performed using the microdomain boundary to find the subset of the synapse centroids included in the respective domain (Fig. 7B). From these, a 60% random subset was selected to match the experimental observations (Reichenbach et al., 2010) (Fig. 7C). The synapse points were used as attractors for the astrocyte morphogenesis algorithms (see section , Fig. 7D).

Following the generation of the astrocyte morphologies, the neuroglial connectivity is updated by finding for each centroid in the synaptic cloud the closest morphological segment in the morphology (Fig. 7E).

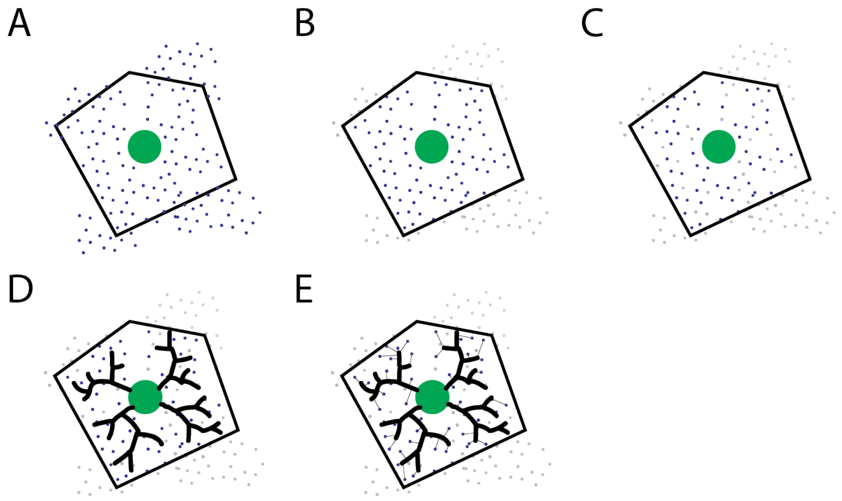

Figure 7 Algorithmic overview of the computation of neuroglial connectivity. (A-B) First the synapse centroids included in each astrocytic microdomain are found. (C) A 60% is selected. (D) The morphology grows, using the synapses as attraction points. (E) For each synapse the closest segment is annotated.

Glialglial

The gap junctions between neighboring astrocytes were determined as touches between neighboring colliding morphologies of the grown astrocytes, using the process of touch-detection as presented in Markram et al. (2015)). For this step, the full-grown astrocyte morphology was required.

1.6 Topological synthesis

Apart from the challenges encountered at the description of neuronal morphologies, astrocytes present some additional obstacles. The extremely limited number of astrocytic reconstructions with sufficient details renders any statistical analysis a challenge by itself. In (Kanari et al., 2020), the authors claim that neurons with less than 5 cells are hard to describe and computationally generate due to lack of sufficient inputs. Yet, we are facing an impossible problem: to extract meaningful descriptions from only three complete reconstructions. A morphological analysis of key morphometrics of the three available reconstructions (Fig. 8) reveals the limitations of the most common synthesis techniques to be applied on astrocytes. The cells present high variance on basic morphometrics, such as number of sections, branch orders and distribution of path and radial distances. As a result, the description of these cells with common population measurements would not be possible as the variance is orders of magnitude higher that the average values.

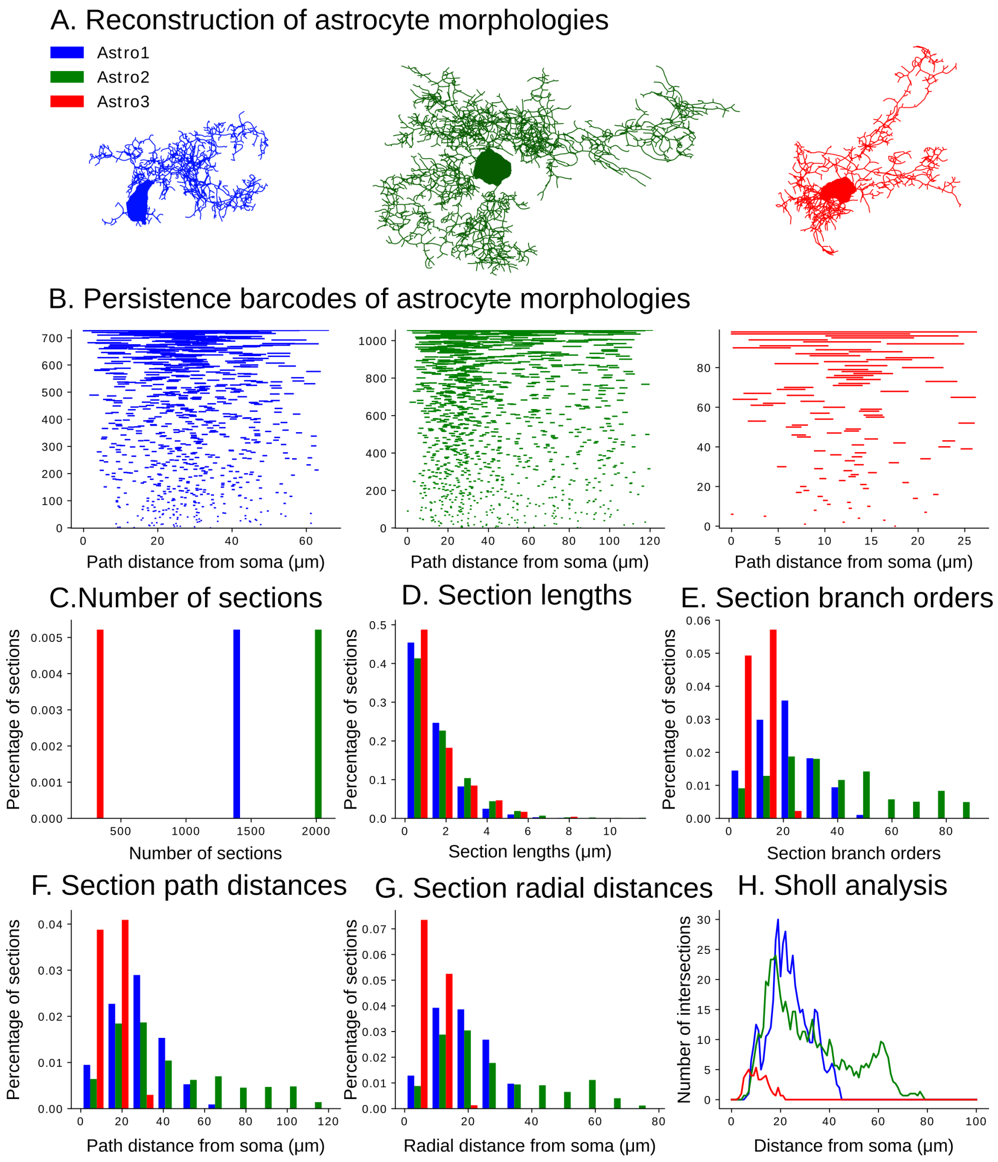

Figure 8 Morphological comparison of astrocyte reconstructions. A. Examples of single cell reconstructions of astrocytes (individual cells illustrated in blue, green, red). B. The corresponding barcodes of the perivascular processes. Basic morphometrics (C-H) of perivascular processes for the three astrocyte reconstructions illustrated in A.

A synthesis algorithm based on the topological properties of a neuron has been proposed by Kanari et al. (2020). It has been shown that the persistence barcode of different neuronal cell types is sufficient to capture the different growth mechanisms that lead to the distinct shapes of dendrites

(Kanari et al., 2020). The topological barcodes of astrocytes(Fig.8B) can capture the key properties of their morphologies. For example, the number of branches is reflected in the number of bars in the barcode (Fig. 8B and C) and the path and radial distances in the extends of the branches (Fig. 8B and F). Therefore, TMD is a useful descriptor for the complex morphologies of astrocytes. Can this technique be used to synthesize cells with similar morphological properties to the population of reconstructed morphologies?

The TMD of astrocytes is used to define the bifurcation and termination probabilities of each branch during synthesis; the coupling of these probabilities provides a method to implicitly reproduce key correlations between morphological features. In addition to the TMD, we need to extract some morphological features that are not encoded in the branching topology of the tree such as soma size, trunk orientation, and thickness of branches.

Space colonization terminology

A space colonization strategy (Runions et al., 2005, 2007), grows tree structures that are embedded in space competing for resources, in a similar fashion to the work of Cuntz (Cuntz et al., 2010). The main difference between these two approaches is that in the case of the space colonization algorithm the tree grows its points towards the average of the nearest attraction points (resources), instead of using them as nodes of the tree itself. Summarizing the space colonization steps: In each iteration, for each node the attraction points that are closer than an influence distance d_i are found. For each node a new direction is calculated from the average of the unit vectors to its closest points and a new point is added. Once the points are created, the attraction points that are closer to the tree nodes than kill distance d_k are removed.

The tree growing approach of the space colonization algorithm is conceptually compatible with the growing of stochastic trees, allowing for a more natural addition to TNS for encompassing spatial embedding. However, given that the algorithm generates multiple points and by extension multiple branches at each step, a major restructuring is still required, so that it is possible to work with segment by segment grown binary trees.

The influence and kill distances, which depend on the segment length L, are defined:

$$d_k = \alpha_k L d_i = \alpha_i L \tag{8}$$

where, α_k, α_i are the influence and kill factors respectively. Astrocyte synthesis employs a variable segment length, therefore the aforementioned factors are the parameters that will be chosen, while the length distribution is determined from the tree barcodes (see section 1.6).

The synaptic point cloud for each astrocyte is determined during the neuroglial generation stage of the NGV circuit building. Thus, each astrocyte has access to a set of attraction points, which will be used as the point cloud for the space colonization component of astrocyte synthesis.

Initiation of processes on soma

The first step of growing virtual trees is to calculate the starting point x_0 on the surface of the soma, which is represented as a sphere. During cell placement the position x_s and radius r_s of each astrocyte is calculated, and endfeet targets are generated during the gliovascular connectivity step. The microdomain tessellation is also required because it reflects the extents of each cell. Thus, each microdomain is available as a set of points and triangles.

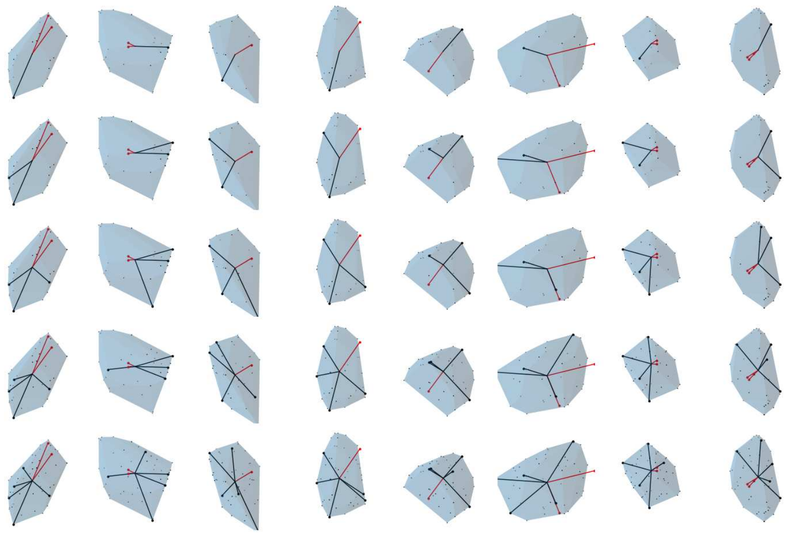

Figure 9 Examples of domain orientation for varying numbers of perivascular (red) and perisynaptic (black) processes.

Perivascular initial points are created first because of their dependence on the endfeet targets, which are already predetermined. An endfoot target x_t is assigned to each perivascular tree, the initial point of which is calculated:

$$d_0^{perivascular} = \frac{x_t - x_s}{\|x_t - x_s\|} \tag{9}$$

$$x_0^{perivascular} = d_0^{perivascular} r_s \tag{10}$$

Sampling from the number of primary process distribution, the total number of processes for the cell is drawn. If the total number is higher than the number of perivascular processes, then the number of perisynaptic processes is calculated from their difference. The goal for estimating the initial points for perisynaptic trees is twofold: evenly distributing the points so that the trees don't overlap and influencing their location with respect to the microdomain anisotropy. The microdomain triangles are barycentrically subdivided the number of vertices is increased tenfold, creating a dense sampling of points on the surface of the domain. The vectors from the soma center to the vertices of the triangles, that are created from the subdivision process on the domain surface, constitute the orientation vectors $V = \{ v_1, v_2, ..., v_n \}, v_i \in R^3$, from which the perisynaptic orientations will be chosen. The set of chosen orientations C is initially populated with the endfeet orientations from the previous. At each iteration a perisynaptic orientation is estimated by maximizing the cost function below and is added to C.

$$v = \arg\max_{v \in (V - C)} J(v) \tag{11}$$

$$J(v) = (1 - \alpha) \frac{\sum_{v_k \in C} \theta(v, v_k)}{|C|} + \alpha \frac{\|v\|}{l_{max}} \tag{12}$$

$$\theta(v, u) = \frac{1}{\pi} \arccos{(v \cdot u)} \tag{13}$$

where α is the mixing factor and chosen to be 0.05, l_{max} is the maximum length of the vectors in V, and $\theta(v, u)$ the normalized squared angle function. The selection of the mixing factor prioritizes equidistribution over length bias, so that orientations are evenly distributed apart from the longest vectors that influence significantly the orientation choice. The process continues until all orientations are calculated and each perisynaptic point are calculated:

$$d_0^{perisynaptic} = \frac{v}{\|v\|} \tag{14}$$

$$x_0^{perisynaptic} = d_0^{perisynaptic} r_s \tag{15}$$

In figure 9, examples of orientations can be seen for varying numbers of perivascular and perisynaptic processes.

Elongation

Elongation is the process responsible from the creation of consecutive points that grow a tree. A pair of points that is created defines a segment, the length of which is sampled from a normal distribution, $L \sim \mathcal{N}(\mu_{seg}, \sigma^2_{seg})$, the mean of which are estimated from the smallest bars in the input barcode of the trees.

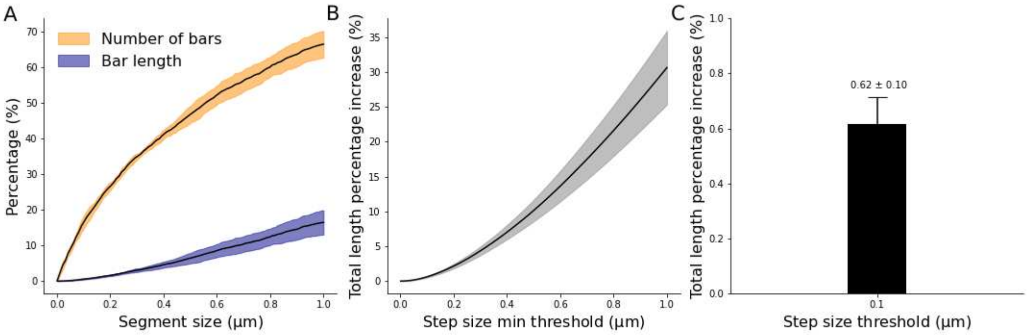

Figure 10 Bar length distributions. (A) Cumulative percentage of persistence bars that are smaller than segment size. (B) Total length increase in morphology with respect to minimum segment length and the exact value for segment length of 0.1 µm

In order to estimate the mean segment length, the cumulative percentage of the number of bars and bar lengths that are smaller than the segment size was plotted (Fig. 10A). Only a small fraction of bars (<10%) were smaller that 0.1 µm, indicating a good choice for the segment length threshold. The goal of this curation process isn't the removal of the smaller bars as it would remove branching points altering the topology, but rather the scaling of the bars below the threshold. The effect of the bar scaling was investigated in figure 10B, where the total length change of the morphology is plotted with respect to the chosen threshold and shows a negligible increase in total length which was quantified as (0.6 ± 0.1)µm (Fig. 10C). A small $\sigma_{seg} = 0.001$ was chosen to allow for a small amount of variability.

A segment also assumes a direction D_{seg}, which is a weighted sum of different contributions, giving rise to the phenomenological behavior of the cell growth. In previous work focusing on neuronal synthesis (Kanari et al., 2020; Koene et al., 2009) the direction of the segment is a weighted sum of three unit vectors: the cumulative memory M, a random vector R and a target vector

T. Cumulative memory sums all previous directions, exponentially decreasing the contributions so that previous directions far in the past are quickly forgotten. The targeting vector T is calculated during branch splitting and remains unchanged during elongation.

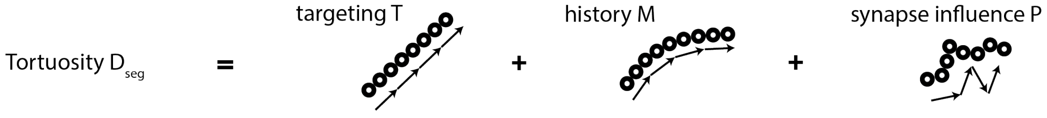

Figure 11 Section tortuosity is a combination of three contributions in astrocyte synthesis: targeting, history and influence from the point cloud

In this work, the stochastic interaction with the environment is not captured by a random contribution, sampled uniformly from the unit sphere, but by the unit direction to the nearest synaptic seed in the point cloud. Therefore, the segment direction, known as tortuosity, takes the form (see Fig. 11):

$$D_{seg} = \tau T + \mu M + \rho P \qquad \tau + \mu + \rho = 1 \tag{16}$$

The interplay between targeting τ and randomness ρ factors determine the degree of tortuosity of the processes. All parameters take values from the interval $[0, 1]$, therefore a configuration, for example, of $(\tau, \mu, \rho) = (1, 0, 0)$ would produce straight processes that follow the initial branching direction and exhibit no tortuosity. On the other hand, a configuration $(\tau, \mu, \rho) = (0, 0, 1)$ would generate processes the direction of which will be exclusively dependent on the closest synaptic seed. The history contribution induces a rigidity effect to the growth of the processes that reflects their intrinsic structural properties.

Following the calculation of the new direction a new point is created if it doesn't collide with the microdomain boundary. If it does collide a termination signal is sent and the elongation is stopped for the specific process. Otherwise, all the synaptic points that are closer to the new point than a kill distance $d_k = \alpha_k L$ are removed from the point cloud. The astrocyte synthesis algorithm grows the morphology trees in turn, growing by one segment each tree sequentially before growing the next segment from the same tree. This ordering of growth ensures that all trees grow simultaneously completing for the same resources, instead of favoring the growth of the tree that grows first.

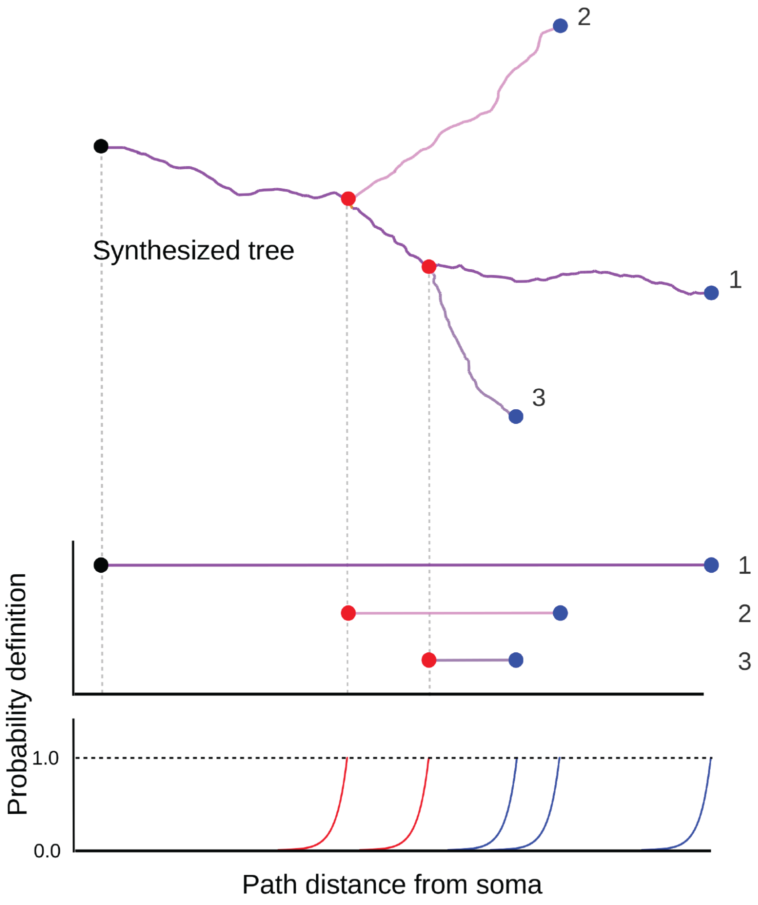

Figure 12 Topological branching and termination based on TMD probabilities. Example of a tree (on top) that is synthesized, bifurcations are annotated in red, terminations annotated in blue. On the bottom the corresponding persistence barcode defines two independent probabilities (to bifurcate in red, to terminate in blue) that the growth algorithm respects during synthesis.

1.7 Branching and termination

TMD dependent probabilities

The basic principle for the tree growth can be summarized by the following steps: the tree has a probability to bifurcate and to terminate associated to the path distance from the soma 12. During the growth process, the topological barcode, which is extracted from the reconstructed morphology population, determines these probabilities to terminate or bifurcate. Similarly to neuronal synthesis, the bifurcation / termination probabilities depend ex-

ponentially on the path distance of the growing tip from the soma 12. This means that when the growing tip approaches the target bifurcation or termination distances as defined from the barcode, the probability to bifurcate or terminate increases exponentially until it reaches 1 after the target distance is surpassed.

Branching of astrocytes

Each growing branch of the astrocyte is assigned one of the following three "growing" types: major, secondary and endfoot. These types are essential to the growing process, because they allow different behaviors to be captured. Major sections represent the primary processes of the astrocytes that initially grow radially outwards and then ramify into the secondary branches. The first section that is generated from the initiation process out of the soma (see section 1.6), is always assigned the major type. Let a growing tip x of a branch with targeting direction $\hat{d}$ and a growing type, which encounters a splitting event mandated by the barcode. The aim of the splitting algorithm is to generate the the targeting directions $\hat{d}_1$, $\hat{d}_2$ and growing types for the children branches.

The splitting algorithm for perisynaptic processes utilizes two of the three growing types: major and secondary. The children sections receive a (major, secondary) pair if the parent is major or a (secondary, secondary) pair otherwise. The procedure uses the mean segment length μ_{seg} to determine the kill $d_k = \alpha_k \mu_{seg}$ and influence $d_i = \alpha_i \mu_{seg}$ distances, which are ubiquitous in the spatial queries around the growing tip. The repulsion points $P_R(x)$ around the tip x are defined as the subset of the morphology points P_M that are closer to x than the kill distance d_k:

$$P_R(x) = \{ \, p \mid \|p - x\| \leq d_k \, \forall \, p \in P_M \, \} \tag{17}$$

If $P_R(x)$ is not empty, the repulsion vector is calculated by averaging the unit directions multiplied with an exponentially decaying contribution that is a function of their respective lengths:

$$\vec{r}(x) = \frac{1}{|P_R(x)|} \sum_{p \in P_R(x)} \frac{p - x}{\|p - x\|} e^{-\frac{\|p-x\|}{d_k}} \tag{18}$$

The repulsion contribution has a decay rate $\frac{1}{d_k}$ (see figure 13), utilizing the kill distance instead of an extra parameter, which makes a reasonable choice while reducing the parameter space.

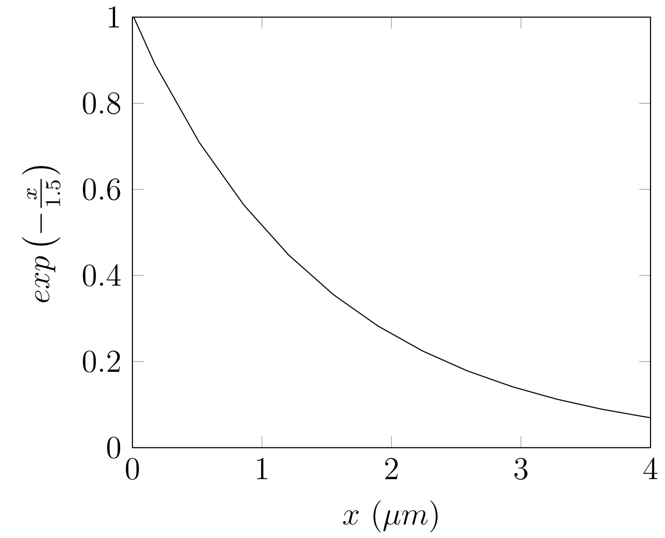

Figure 13 Example of repulsion contribution in the distance interval $[0, 4]$ for a kill distance $d_k = 1.5$ μm

The calculation of the first of the two child unit directions $\hat{d_1}$ depends on the growing type of the current section. If the current section has major type then $\hat{d_1}$ is the normalized sum of the branch direction $\hat{d}$ and the repulsion vector $\vec{r}(x)$:

$$\hat{d_1} = \frac{\hat{d} - \vec{r}(x)}{\|\hat{d} - \vec{r}(x)\|} \tag{19}$$

If the parent is a secondary section, first the set $P_A(x)$ of attraction points in the tip proximity are determined as the points in the synaptic point cloud P_S that are closer to the tip x than the influence distance d_i:

$$P_A(x) = \{\, p \mid \|p - x\| \leq d_i \, \forall \, p \in P_S \,\} \tag{20}$$

and the first direction is calculated as the direction to the closest attraction point s.

$$s = \underset{s \in P_A(x)}{\arg\min} \, \|s - x - \vec{r}(x)\| \tag{21}$$

$$\hat{d_1} = \frac{s - x - \vec{r}(x)}{\|s - x - \vec{r}(x)\|} \tag{22}$$

The second direction $\hat{d}_2$ is always calculated as the direction to the seed that is at the largest angle with the first direction $\hat{d}_1$.

$$s = \underset{s \in P_S(x)}{\arg\min} \left(\frac{s - x - \vec{r}(x)}{\|s - x - \vec{r}(x)\|} \cdot \hat{d}_1 \right) \tag{23}$$

$$\hat{d}_2 = \frac{s - x - \vec{r}(x)}{\|s - x - \vec{r}(x)\|} \tag{24}$$

The splitting algorithm for perivascular trees is an extension of the perisynaptic strategy with the addition of the attraction mechanics to the endfeet targets. Each tree is assigned an endfoot target x_t, which is initially active. An active target attracts major section processes of the perivascular tree until the target is reached. A bias is added to the direction $\hat{d}_1$ of the major processes:

$$\hat{d}_t = \frac{x_t - x}{\|x_t - x\|} \tag{25}$$

$$\hat{d}'_1 = \frac{\alpha \hat{d}_t + (1 - \alpha)\hat{d}_1}{\|\alpha \hat{d}_t + (1 - \alpha)\hat{d}_1\|} \tag{26}$$

where α is the target proximity factor, which depends on the ratio of the distance from the soma to the endfoot target and the distance from the growing tip to the endfoot target.

If a growing tip x is in proximity of the endfoot target, then $\hat{d}_2$ is replaced by the direction to the target $\hat{d}_t$ and that child section is assigned the endfoot growing type which has the sole responsibility to grow towards the endfoot point.

1.8 Vasculature attraction field analysis

In order to model the chemo-attractive field which influences the growth of the perivascular processes, we need first to make some assumptions of its form and properties: We assume that there isn't a preferred direction of the diffusive gradient of the chemo-attractive molecules , i.e. it's isotropic. Furthermore, given that the vasculature graph and surface mesh are not available at the time of the morphology generation , the second assumption constrains the field to be generated by a point source instead of the entire surface of the vasculature in the vicinity.

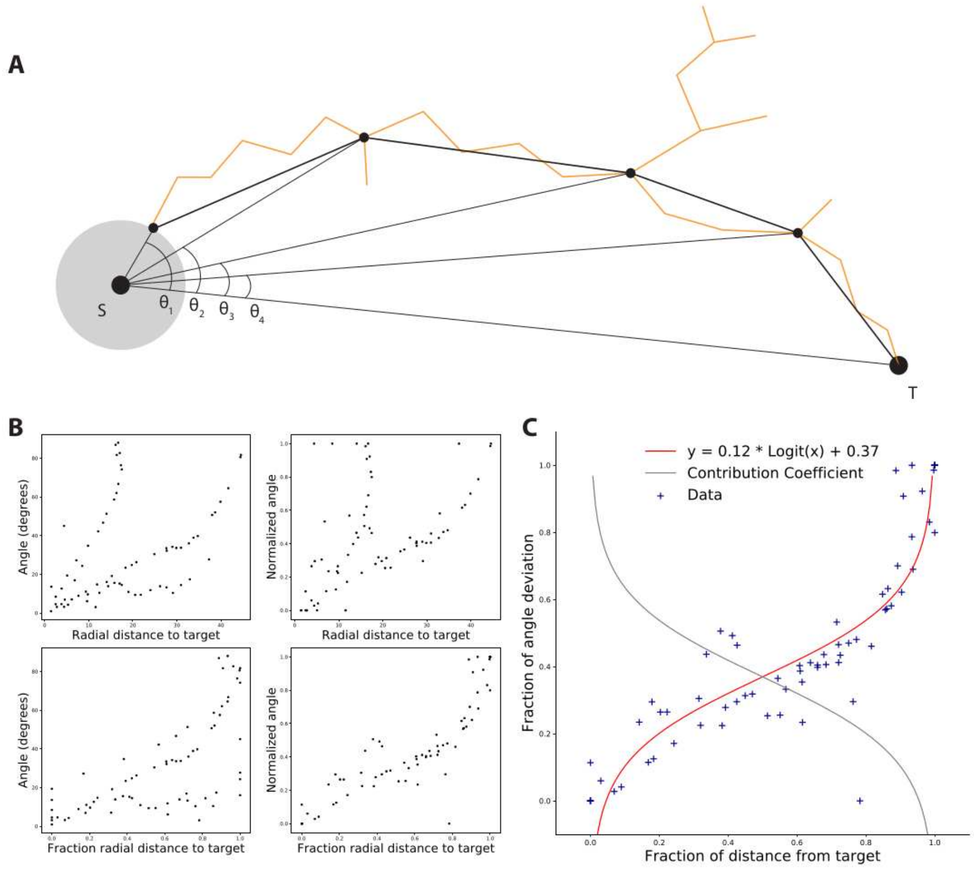

Figure 14 Attraction field analysis. (A) Analysis of radial distances from the soma S to each section end and the respective angle to $\vec{ST}$. (B) Scatter plots of non-normalized and min-max normalized data. (C) min-max normalized data (blue scatter) of how influenced the direction of the process is as it approaches the target along with the logit function fit (red) and the corresponding contribution coefficient α

For each reconstructed astrocyte the endfeet targets were annotated as points close the the termination of an endfoot process. The closest leaf was found for each endfoot target and the upstream sections from the leaf to the root were extracted as show in Figure 14A.

Let a point T be the target point, S the center of the astrocytic soma. For each first point p_i of each section, the angles $theta_i$ between $\vec{SP_i}$ and ST, as a function to the radial distance to the target TP_i. However, measurements on different trees lead to different trends that depend on the extent and orientation of the tree inside the attraction field. In order to normalize the data and quantify the underlying attraction trend we performed min-max

normalization for both angles and radial distances as shown in the comparison of figure 14B.

The attraction of the main process to the target was fit using the quantile logit function (Figure 14C).

$$y(x) = 0.12 \times L(x) + 0.37 \qquad x \in [0,1] \tag{27}$$

$$L(x) = \ln\left(\frac{x}{1-x}\right) \tag{28}$$

The function $a(x)$ produces values in the $(-\infty, \infty)$, but we are only interested in the interval $[0, 1]$. For this reason we introduce the clamp function $c(x) = \max(0, (\min(x, 1)))$, which limits the image into the desired interval. Finally, let d_t and d_{s_i} be the distances from the soma center to the target. The contribution factor of the direction to the target can be calculated, by substituting x with $1 - x$ and by using the identity $logit(1 - x) = -logit(x)$:

$$a\left(\frac{d_{s_i}}{d_t}\right) = c\left(y\left(1 - \frac{d_{s_i}}{d_t}\right)\right) = c\left(-0.12 \times L\left(\frac{d_{s_i}}{d_t}\right) + 0.37\right) \tag{29}$$

1.9 Surface area and volume distribution

Due to a high surface-to-volume ratio in astrocytes processes, it is difficult to capture their membrane geometry using a cylinder representation. For this reason, the volume and surface of segments are separately encoded in their diameters and perimeters respectively. The model for generating synthetic diameters for astrocytes is based on the neuronal algorithm (Kanari et al., 2020). First, distributions of the model parameters are obtained from a fit to the available reconstructed astrocytes. Then, diameters are generated using parameters sampled from these distributions. The model parameters include the sibling ratio (ratio between diameters of daughter branches), diameter power relation (to model the relative diameters between parent and daughter branches), taper rates, trunk and terminal diameters. See for example (Ascoli et al., 2008) for more details one these parameters. We deviate from the neuronal diameter model by allowing for negative taper rates, to obtain increasing and decreasing diameters with path distances. Along the neurites, diameters are bound by the trunk and terminal diameters, sampled for each neurite, while the other parameters are sampled at each segment or bifurcation.

Regarding the distribution of perimeters on the synthesized astrocytes, we extracted the diameter-perimeter pair values from the digital reconstructions and fit a linear regression model:

$$P_i = \beta_0 + \beta_1 D_i + \epsilon_i \tag{30}$$

where P_i, D_i are the perimeter and diameter respectively and ϵ_i the variation or noise in the data. Thus, following the diametrization process we assigned the perimeters using the linear predictor function shown above.

1.10 Endfeet surface reconstruction & pruning

The growth of one endfoot was modelled as the solution $t(x)$ to the eikonal equation:

$$\begin{aligned} |\nabla_S t(\mathbf{x})| &= \frac{1}{f(\mathbf{x})},\ \forall \mathbf{x} \in \mathcal{S} \subset \mathbb{R}^3 \\ \phi(x) &= 0,\ \forall \mathbf{x} \in \partial S \end{aligned} \tag{31}$$

which is first-order partial differential equation, where S is the vasculature surface, a 2D smooth and closed manifold in $\mathbb{R}^3$, ∇_S is the gradient in the tangent plane to the manifold, $t(x)$ is the distance or travel time from the source and $f(\mathbf{x})$ is the speed of travel. Thus, $t(x)$ provides the time that an interface (contour) will need to reach $\mathbf{x}$ from the initial location ∂S. We are particularly interested in the simplified form where $f(\mathbf{x}) = 1$ and equation 31 is converted to the signed distance function from the boundary ∂S.

In our use case, in which we want to model the growth of an endfoot on the vasculature manifold, the boundary ∂S corresponds to the endfoot target $\mathbf{x}_e$. Thus, the eikonal equation gives the travel times from the endfoot target to any point on S along the geodesics of its surface. Generalizing this to multiple endfeet targets, would required to calculate the travel times from each surface point to each endfoot target.

To approximate the solution of 31 on triangulation S_T of the surface S, which is comprised of nodes x_i, I implemented the fast marching method for triangulated surfaces (Fu et al., 2011). Each node is assigned a value T_i that corresponds to its travel time, which are initially set to $+\inf$ except for the endfoot's one which is 0. Using the one-ring neighbors for each node, the approximated solution of the travel T_i at node x_i is calculated from as the minimum of shortest path distances calculated from all the triangles in then neighborhood.For a triangle (v_1, v_2, v_3), if v_1 and v_2 are upwind of v_3, there is a characteristic line of the gradient $\nabla_S t(\mathbf{x})$ that passes from v_3 and crosses the base of the triangle $\vec{e}_{1,2} = v_2 - v_1$ at x_λ. Thus, the travel time at T_3 is given by:

$$\begin{aligned} T_3 &= T_\lambda + T_{\lambda,3} \\ T_3 &= T_1 + \lambda\,(T_2 - T_1) + \|\vec{e}_{1,3} - \lambda \vec{e}_{1,2}\| \end{aligned} \tag{32}$$

which is derived from the fact that the approximation is linear, thus $T_\lambda = T_1 + \lambda T_{1,2}$ and $T_{\lambda,3} = f\|\vec{e}_{\lambda,3}\| = \|\vec{e}_{\lambda,3}\|$, because we have set the speed function to 1. To represent the gradient characteristic, λ should minimize T_3 and must be in the range $[0, 1]$.

In order to introduce the notion of multiple endfeet growing in parallel, each node x_i on S_T was assigned an endfoot group G_i. Thus, upon initialization all endfoot nodes are assigned $T_i = 0$ and $G_i = i$, where $i = (0, 1, 2, ..., N_{endfeet})$. A priority queue was implemented that allowed to update first the nodes with the smallest travel time at each iteration, simulating the propagation of wavefronts. As nodes were updated with the shortest travel time to the nearest endfoot nodes, the group label of that endfoot was assigned to them and if the node had already a group assigned, the update stopped. This allowed the propagation of the "endfoot waves" on S_T competitively as they were allows to propagate to nodes that were not already captured from a neighbor. The algorithm finished when there were no more vertices in the priority queue to update.

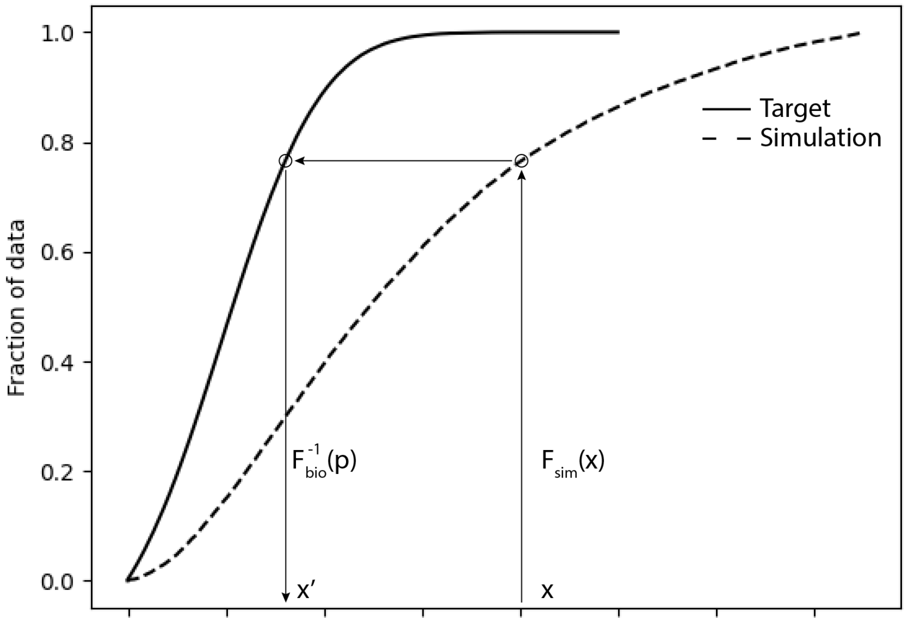

Figure 15 Diagram of the are transformation from the simulation area distribution to the target one extracted from the literature via the inverse CDF transform.

The endfeet meshes were reconstructed from the nodes in each group G_i. Due to the fact that the areas of the endfeet covered almost entirely the

vasculature surface, a pruning procedure was introduced in order to match a target area distribution, extracted from the literature. Thus, given a target cumulative distribution F_{bio} and the empirical distribution from the simulation $\hat{F}_{sim}$, overshoot endfeet areas A_i were transformed into the respective target ones A'_i via the cumulative inverse transform:

$$A'_i = F_{bio}^{-1}\left(\hat{F}_{sim}(A_i)\right) \tag{33}$$

To match the new pruned areas, the geometry of the endfoot meshes was pruned using the travel times that were calculated in the previous step. For each triangle in the endfoot mesh the average travel time was calculated from its vertices and the triangles with the highest travel times were removed one by one until the target area was reached. In other words, the meshes shrunk, starting from the periphery until the target area was approximately reached.

2 Supplementary Figures

2.1 Endfeet morphometrics

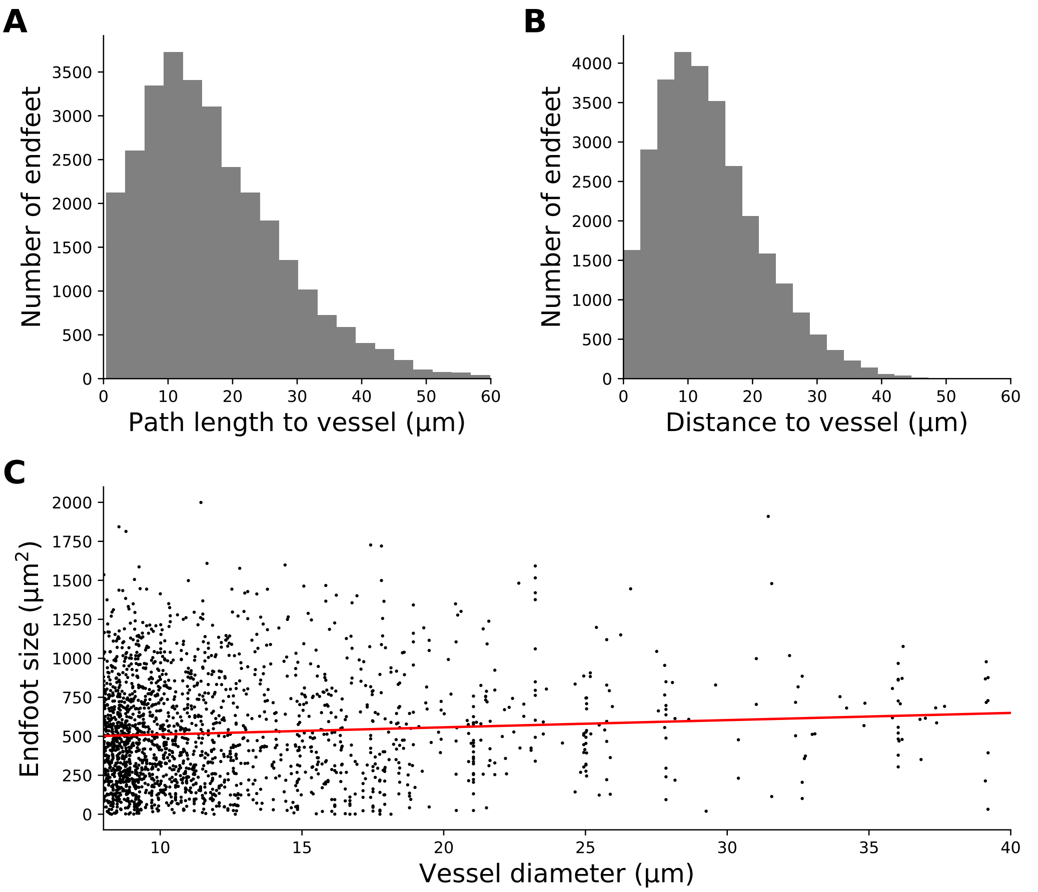

Figure 16 Morphometric analysis of perivascular processes path length and distance to the vascular site and the relationship between endfoot size and vessel diameter. (A) Histogram of perivascular processes' path length, starting at the soma and ending at the vascular site, where the endfoot has been formed. (B) Histogram of perivascular processes' euclidean distance, between the soma surface and the vascular site. (C) Vessels exhibited increasing endfoot areas with increasing vessel diameters.

The path and Euclidean distances were quantified for each perivascular endfoot in the astrocytic population (Figure 16A-B). Path length was calculated from the soma and along the shortest path to the vascular site, whereas the euclidean distance was calculated from the surface of the soma to the vascular site. The path length distribution exhibited a mean of (16 ± 10) µm, a median of 14.9 µm, a 5th percentile of 2.6 µm and a 95th percentile of 37.6 µm. The euclidean distance distribution exhibited a mean of (13 ± 8) µm, a median of 12.1 µm, a 5th percentile of 2.5 µm and a 95th percentile of 28.5 µm.

We measured the surface area for each endfoot and the respective vessel diameter. We found that for increasing vessel diameters the endfoot size increased (Figure 16C, $p < .0001$, slope=4.6).

3 Supplementary Tables

Animal	Region	Age	Staining Method	Mean (mm^{-3})	Std (mm^{-3})	Citation
Rat	Neocortex	P13-P28	SR101	17900	1400	Nimmerjahn et al. (2004)
Mouse	Neocortex	P32-P272	SR101	14200	1100	Nimmerjahn et al. (2004)
Mouse	Neocortex	Neonate	S100b	2666	133	Emsley and Macklis (2006)
Mouse	Neocortex	Neonate	GFAP	333	33	Emsley and Macklis (2006)
Mouse	Neocortex	Adult	S100b	15696	860	Grosche et al. (2013)
Mouse	Neocortex	Old	S100b	16490	872	Grosche et al. (2013)
Mouse	Hippocampus	Adult	S100b	20904	1924	Grosche et al. (2013)
Rat	Neocortex	P56	SR101	18000	2000	Leahy et al. (2013)
Rat	Neocortex	Juvenile	GFAP	10700	1750	Olude et al. (2015)
Rat	Neocortex	Adult	GFAP	7075	920	Olude et al. (2015)
Mouse	Hippocampus	P14	GLT-1, GLAST	10800	400	Schreiner et al. (2014)

Table 1 Summary of experimentally measured values of average astrocytic densities

Total length (m)	L1	L2	L3	L4	L5
Neurons	169.6	157.5	406.7	271.6	636.7
Astrocytes	10.3	8.6	20.6	10.7	29.8
Vasculature	0.2	0.2	0.3	0.2	0.4
Length density (m/mm^3)	L1	L2	L3	L4	L5
Neurons	1308.9	1347.7	1467.2	1824.4	1541.4
Astrocytes	79.2	73.7	74.2	71.5	72.3
Vasculature	1.3	1.4	1.2	1.4	0.9
Total surface area (mm^2)	L1	L2	L3	L4	L5
Neurons	242.9	188.8	482.1	335.9	781.7
Astrocytes	31.0	26.0	62.2	32.1	90.0
Vasculature	3.2	3.2	6.1	3.2	6.2
Surface area density (mm^2/mm^3)	L1	L2	L3	L4	L5
Neurons	1874.9	1615.4	1739.4	2256.2	1895.4
Astrocytes	239.3	222.4	224.2	215.8	218.3
Vasculature	25.0	27.2	21.9	21.8	15.0
Total volume ($\times 10^{-2}$ mm^3)	L1	L2	L3	L4	L5
Neurons	4.8	3.6	9.8	7.4	16.0
Astrocytes	0.6	0.5	1.1	0.6	1.6
Vasculature	0.6	0.6	1.0	0.5	0.9
Volume occupancy (% of the layer volume)	L1	L2	L3	L4	L5
Neurons	37.2	30.5	35.5	49.4	38.8
Astrocytes	4.3	4.0	4.0	3.9	3.9
Vasculature	4.7	5.0	3.9	3.2	2.2

Table 2 Quantification of the total wiring, total surface areas and volume fractions for neurons, astrocytes and the vasculature and for each layer in the NGV circuit.

Measurement	
	Median
Total endfeet area per astrocyte (μm^2)	427
Total endfeet volume per astrocyte (μm^3)	414
Gap junctional connections	198
Number of synapses per astrocyte	3010
	Mean $\pm$ Std
Neurons per astrocyte	627 $\pm$ 259
Neuronal somata per astrocyte	6 $\pm$ 4
Neighboring astrocytes per astrocyte	5 $\pm$ 2
	Range
Neuronal wiring per domain (m)	0.012 - 0.218
Neuronal segment area per domain (mm^2)	0.050 - 0.275
Neuronal segment volume per domain ($\times 10^6\ \mu m^3$)	0.003 - 0.061

Table 3 Quantification of astrocytic related numbers, extracted from the NGV circuit.

References

Abdellah M, Guerrero NR, Lapere S, Coggan JS, Keller D, Coste B, Dagar S, Courcol JD, Markram H, Schürmann F. 2020. Interactive visualization and analysis of morphological skeletons of brain vasculature networks with VessMorphoVis. Bioinformatics. 36:i534–i541. Publisher: Oxford Academic.

Appaix F, Girod S, Boisseau S, Römer J, Vial JC, Albrieux M, Maurin M, Depaulis A, Guillemain I, Sanden Bvd. 2012. Specific In Vivo Staining of Astrocytes in the Whole Brain after Intravenous Injection of Sulforhodamine Dyes. PLOS ONE. 7:e35169.

Ascoli GA, Alonso-Nanclares L, Anderson SA, Barrionuevo G, Benavides-Piccione R, Burkhalter A, Buzsáki G, Cauli B, DeFelipe J, Fairén A, Feldmeyer D, Fishell G, Fregnac Y, Freund TF, Gardner D, Gardner EP, Goldberg JH, Helmstaedter M, Hestrin S, Karube F, Kisvárday ZF, Lambolez B, Lewis DA, Marin O, Markram H, Muñoz A, Packer A, Petersen CCH, Rockland KS, Rossier J, Rudy B, Somogyi P, Staiger JF, Tamas G, Thomson AM, Toledo-Rodriguez M, Wang Y, West DC, Yuste R, The Petilla Interneuron Nomenclature Group (PING). 2008. Petilla terminology: nomenclature of features of GABAergic interneurons of the cerebral cortex. Nature Reviews Neuroscience. 9:557–568. Number: 7 Publisher: Nature Publishing Group.

Beckmann N, Kriegel HP, Schneider R, Seeger B. 1990. The R*-tree: an efficient and robust access method for points and rectangles. In: Proceedings of the 1990 ACM SIGMOD international conference on Management of data. SIGMOD '90. New York, NY, USA: Association for Computing Machinery. p. 322–331. doi:10.1145/93597.98741.

Calì C, Agus M, Kare K, Boges DJ, Lehväslaiho H, Hadwiger M, Magistretti PJ. 2019. 3D cellular reconstruction of cortical glia and parenchymal morphometric analysis from Serial Block-Face Electron Microscopy of juvenile rat. Progress in Neurobiology. 183:101696.

Cuntz H, Forstner F, Borst A, Häusser M. 2010. One Rule to Grow Them All: A General Theory of Neuronal Branching and Its Practical Application. PLoS Computational Biology. 6.

Dai K, Hernando J, Billeh YN, Gratiy SL, Planas J, Davison AP, Dura-Bernal S, Gleeson P, Devresse A, Dichter BK, Gevaert M, King JG, Geit WAHV, Povolotsky AV, Muller E, Courcol JD, Arkhipov A. 2020. The SONATA

data format for efficient description of large-scale network models. PLOS Computational Biology. 16:e1007696. Publisher: Public Library of Science.

Dereudre D. 2019. Introduction to the theory of Gibbs point processes. In: Stochastic Geometry. Springer. p. 181–229.

Emsley JG, Macklis JD. 2006. Astroglial heterogeneity closely reflects the neuronal-defined anatomy of the adult murine CNS. Neuron glia biology. 2:175. Publisher: NIH Public Access.

Erö C, Gewaltig MO, Keller D, Markram H. 2018. A Cell Atlas for the Mouse Brain. Frontiers in Neuroinformatics. 12.

Fu Z, Jeong WK, Pan Y, Kirby RM, Whitaker RT. 2011. A fast iterative method for solving the eikonal equation on triangulated surfaces. SIAM Journal on Scientific Computing. 33:2468–2488. Publisher: SIAM.

Grosche A, Grosche J, Tackenberg M, Scheller D, Gerstner G, Gumprecht A, Pannicke T, Hirrlinger PG, Wilhelmsson U, Hüttmann K. 2013. Versatile and simple approach to determine astrocyte territories in mouse neocortex and hippocampus. PLoS One. 8:e69143. Publisher: Public Library of Science.

Kacem K, Lacombe P, Seylaz J, Bonvento G. 1998. Structural organization of the perivascular astrocyte endfeet and their relationship with the endothelial glucose transporter: A confocal microscopy study. Glia. 23:1–10. _eprint: https://onlinelibrary.wiley.com/doi/pdf/10.1002/%28SICI%291098-1136%28199805%2923%3A1%3C1%3A%3AAID-GLIA1%3E3.0.CO%3B2-B.

Kanari L, Dictus H, Chalimourda A, Geit WV, Coste B, Shillcock J, Hess K, Markram H. 2020. Computational synthesis of cortical dendritic morphologies. bioRxiv. :2020.04.15.040410Publisher: Cold Spring Harbor Laboratory Section: New Results.

Koene RA, Tijms B, van Hees P, Postma F, de Ridder A, Ramakers GJ, van Pelt J, van Ooyen A. 2009. NETMORPH: a framework for the stochastic generation of large scale neuronal networks with realistic neuron morphologies. Neuroinformatics. 7:195–210. Publisher: Springer.

Köster J, Rahmann S. 2012. Snakemake—a scalable bioinformatics workflow engine. Bioinformatics. 28:2520–2522. Publisher: Oxford Academic.

Leahy C, Radhakrishnan H, Srinivasan VJ. 2013. Volumetric imaging and

quantification of cytoarchitecture and myeloarchitecture with intrinsic scattering contrast. Biomedical Optics Express. 4:1978–1990.

Markram H, Muller E, Ramaswamy S, Reimann MW, Abdellah M, Sanchez CA, Ailamaki A, Alonso-Nanclares L, Antille N, Arsever S, Kahou GAA, Berger TK, Bilgili A, Buncic N, Chalimourda A, Chindemi G, Courcol JD, Delalondre F, Delattre V, Druckmann S, Dumusc R, Dynes J, Eilemann S, Gal E, Gevaert ME, Ghobril JP, Gidon A, Graham JW, Gupta A, Haenel V, Hay E, Heinis T, Hernando JB, Hines M, Kanari L, Keller D, Kenyon J, Khazen G, Kim Y, King JG, Kisvarday Z, Kumbhar P, Lasserre S, Le Bé JV, Magalhães BRC, Merchán-Pérez A, Meystre J, Morrice BR, Muller J, Muñoz-Céspedes A, Muralidhar S, Muthurasa K, Nachbaur D, Newton TH, Nolte M, Ovcharenko A, Palacios J, Pastor L, Perin R, Ranjan R, Riachi I, Rodríguez JR, Riquelme JL, Rössert C, Sfyrakis K, Shi Y, Shillcock JC, Silberberg G, Silva R, Tauheed F, Telefont M, Toledo-Rodriguez M, Tränkler T, Van Geit W, Díaz JV, Walker R, Wang Y, Zaninetta SM, DeFelipe J, Hill SL, Segev I, Schürmann F. 2015. Reconstruction and Simulation of Neocortical Microcircuitry. Cell. 163:456–492.

McCaslin AF, Chen BR, Radosevich AJ, Cauli B, Hillman EM. 2011. in vivo 3D Morphology of Astrocyte—Vasculature Interactions in the Somatosensory Cortex: Implications for Neurovascular Coupling. Journal of cerebral blood flow & metabolism. 31:795–806. Publisher: SAGE Publications Sage UK: London, England.

Moye SL, Diaz-Castro B, Gangwani MR, Khakh BS. 2019. Visualizing Astrocyte Morphology Using Lucifer Yellow Iontophoresis. JoVE (Journal of Visualized Experiments). :e60225.

Nimmerjahn A, Kirchhoff F, Kerr JND, Helmchen F. 2004. Sulforhodamine 101 as a specific marker of astroglia in the neocortex in vivo. Nature Methods. 1:31–37. Number: 1 Publisher: Nature Publishing Group.

Oeltze S, Preim B. 2004. Visualization of anatomic tree structures with convolution surfaces. p. 311–320.

Olude MA, Mustapha OA, Aderounmu OA, Olopade JO, Ihunwo AO. 2015. Astrocyte morphology, heterogeneity, and density in the developing African giant rat (Cricetomys gambianus). Frontiers in neuroanatomy. 9:67. Publisher: Frontiers.

Reichenbach A, Derouiche A, Kirchhoff F. 2010. Morphology and dynamics of perisynaptic glia. Brain Research Reviews. 63:11–25.

Reichold J, Stampanoni M, Keller AL, Buck A, Jenny P, Weber B. 2009.

Vascular Graph Model to Simulate the Cerebral Blood Flow in Realistic Vascular Networks. Journal of Cerebral Blood Flow & Metabolism. 29:1429–1443.

Ruelle D. 1999. Statistical mechanics: Rigorous results. World Scientific.

Runions A, Fuhrer M, Lane B, Federl P, Rolland-Lagan AG, Prusinkiewicz P. 2005. Modeling and Visualization of Leaf Venation Patterns. In: ACM SIGGRAPH 2005 Papers. SIGGRAPH '05. New York, NY, USA: ACM. p. 702–711. doi:10.1145/1186822.1073251.

Runions A, Lane B, Prusinkiewicz P. 2007. Modeling Trees with a Space Colonization Algorithm. In: Proceedings of the Third Eurographics Conference on Natural Phenomena. NPH'07. Aire-la-Ville, Switzerland, Switzerland: Eurographics Association. p. 63–70. doi:10.2312/NPH/NPH07/063-070.

Schreiner AE, Durry S, Aida T, Stock MC, Rüther U, Tanaka K, Rose CR, Kafitz KW. 2014. Laminar and subcellular heterogeneity of GLAST and GLT-1 immunoreactivity in the developing postnatal mouse hippocampus. Journal of Comparative Neurology. 522:204–224. Publisher: Wiley Online Library.
